# Computational Investigation of the Interplay of Substrate Positioning and Reactivity in Catechol O-Methyltransferase

**DOI:** 10.1371/journal.pone.0161868

**Published:** 2016-08-26

**Authors:** Niladri Patra, Efthymios I. Ioannidis, Heather J. Kulik

**Affiliations:** Department of Chemical Engineering, Massachusetts Institute of Technology, Cambridge, Massachusetts, 02139, United States of America; Universidade Nova de Lisboa Instituto de Tecnologia Quimica e Biologica, PORTUGAL

## Abstract

Catechol *O*-methyltransferase (COMT) is a SAM- and Mg^2+^-dependent methyltransferase that regulates neurotransmitters through methylation. Simulations and experiments have identified divergent catecholamine substrate orientations in the COMT active site: molecular dynamics simulations have favored a monodentate coordination of catecholate substrates to the active site Mg^2+^, and crystal structures instead preserve bidentate coordination along with short (2.65 Å) methyl donor-acceptor distances. We carry out longer dynamics (up to 350 ns) to quantify interconversion between bidentate and monodentate binding poses. We provide a systematic determination of the relative free energy of the monodentate and bidentate structures in order to identify whether structural differences alter the nature of the methyl transfer mechanism and source of enzymatic rate enhancement. We demonstrate that the bidentate and monodentate binding modes are close in energy but separated by a 7 kcal/mol free energy barrier. Analysis of interactions in the two binding modes reveals that the driving force for monodentate catecholate orientations in classical molecular dynamics simulations is derived from stronger electrostatic stabilization afforded by alternate Mg^2+^ coordination with strongly charged active site carboxylates. Mixed semi-empirical-classical (SQM/MM) substrate C-O distances (2.7 Å) for the bidentate case are in excellent agreement with COMT X-ray crystal structures, as long as charge transfer between the substrates, Mg^2+^, and surrounding ligands is permitted. SQM/MM free energy barriers for methyl transfer from bidentate and monodentate catecholate configurations are comparable at around 21–22 kcal/mol, in good agreement with experiment (18–19 kcal/mol). Overall, the work suggests that both binding poses are viable for methyl transfer, and accurate descriptions of charge transfer and electrostatics are needed to provide balanced relative barriers when multiple binding poses are accessible, for example in other transferases.

## Introduction

Quantum-mechanical/molecular-mechanics (QM/MM) simulation[1–8] has taken a central role in unraveling enzyme mechanism. Challenges remain in fully enumerating sources of enzymatic rate enhancement even for fundamental reactions such as methyl transfer in neurotransmitter[9] and gene regulation[10, 11]. Catechol *O*-methyltransferase (COMT) is an S-Adenosyl-L-methionine (SAM)- and Mg^2+^-dependent methyltransferase (MTase)[9] that reacts with an array of catecholamine substrates (e.g., dopamine neurotransmitters). In order to form an active Michaelis complex in COMT, SAM binds first, followed by Mg^2+^, and the catecholamine substrate binds last in a bidentate fashion to Mg^2+^ at the solvent-exposed active site[12]. Catechol deprotonation, mediated by a lysine or a histidine in a recently discovered variant[13], has been thought to be an important intermediate step in the catalytic cycle since the catecholate anion is expected to be more reactive[14, 15]. The rate-determining[16], direct S_N_2 methyl transfer[17] from SAM[18] occurs primarily at the meta position[18, 19] of substituted catecholamines, with kinetic studies[12, 20–22] providing a free energy barrier estimate of 18.1[21]-19.2[22] kcal/mol for the soluble, human form of the enzyme. It is believed that Mg^2+^ plays a critical role in bringing the substrates together[18] because complete inhibition of COMT is achieved[9] when Mg^2+^ is replaced by Ca^2+^, and Mg^2+^ may provide a large component of the estimated 10^16^-fold rate enhancement[23] over solution. Alanine mutagenesis of an active site Tyr68 increases the barrier to 21 kcal/mol[22], and combined computational-experimental studies of COMT mutants have suggested[24] enhanced flexibility with respect to the wildtype enzyme is responsible for the reduction in rates.

Atomistic simulation can provide valuable insight into the mechanism by which COMT achieves an estimated 10^16^-fold rate enhancement[23] over solution. Classical molecular dynamics studies should capture motions both at the active site and in the overall protein[25–27], and quantum mechanical studies on model systems of COMT can begin to provide insight into reactivity, typically from static structures[28] due to higher computational cost. Some computational studies have employed classical molecular dynamics (MD) of COMT in apo[29, 30], intermediate[31], and holo[25–27] forms. Long, well-equilibrated MD simulations have only been carried out on apo-COMT[29, 30], and earlier studies on holo-COMT[25–27] were limited by then-available computational power to much shorter 1 ns simulations. Early holo-COMT studies fixed bidentate catecholate coordination to Mg^2+^ observed in X-ray crystal structures[15] with explicit Mg^2+^-O bonds[25, 26]. Although qualitative catecholate-Mg^2+^ coordination was held consistent with experiment, non-bonded SAM methyl-catecholate oxygen (C-O) distances sampled during dynamics averaged 3.55 Å in poor agreement with the approximately 2.6 Å C-O distance observed in crystal structures. Other MD studies[27, 32] in which bidentate catecholate coordination to Mg^2+^ was not enforced instead have resulted in reorientation to form a monodentate catecholate characterized by a single Mg^2+^-O^-^ coordination, an intramolecular hydrogen bond, and a compensating sixth interaction with Mg^2+^ derived from an active site carboxylate. Mixed semi-empirical/classical (SQM/MM) calculations have suggested an even weaker interaction between catecholate and Mg^2+^ with only a single coordination to the neutral hydroxyl of catecholate with a bond elongated by as little as 0.5 Å[33] or as much as 2–5 Å[34] (i.e., catecholate does not coordinate Mg^2+^) with respect to typical Mg^2+^-O bonds. These distinct poses have never before been directly compared, and the substrate's suitability as a methyl acceptor may depend on its position in the active site. For instance, substrate placement has been shown to strongly influence reactivity and branching ratios in metalloenzymes[35, 36].

First-principles simulation of the methyl transfer barrier requires careful selection of which portion of the enzyme will be treated quantum mechanically since QM methods are typically higher scaling and more computationally expensive than classical methods. SAM, catecholamine substrate, and the Mg^2+^ alone are 64 atoms, and the Mg^2+^ coordination sphere enlarges this system size to over 100 atoms. For efficient sampling and calculation, studies have leveraged partial models of SAM and reactants[28] and have often treated Mg^2+^ classically. Despite these approximations, a number of DFT[37, 38] and semi-empirical[32, 39, 40] computational studies[27, 32, 37–40] have produced a wide range of free energy barriers (3–30 kcal/mol) that are sometimes in good agreement with experimental barriers (18–19 kcal/mol), especially after corrections for some approximations. Some of us have identified[24] that large-scale QM/MM treatments (ca. 500 atoms) of the COMT active site 500 atoms in the COMT active site are beneficial for explaining and interpreting the effects of active site mutations. Despite the many approaches that have been proposed in the literature to balance accuracy and efficiency in evaluating methyl transfer barriers, there have not yet been any comparative studies between how differing binding poses accessible in MD may be more or less suitable methyl group acceptors in methyl transfer reactions.

We thus use comprehensive classical and quantum mechanical (both semi-empirical and density functional theory) methods to address open questions in the structure and function of COMT. We quantify the free energy landscape of substrate dynamics, identify the driving force and interactions in differing substrate poses in the active site, and determine the extent to which substrate placement alters the methyl transfer reaction coordinate. We additionally demonstrate that charge transfer between substrates and the active site is required to reproduce experimental crystal structure geometries and methyl transfer barrier heights. The structure of this article is as follows. First, we describe the Computational Details of all simulations carried out in this work. In the Results and Discussion, we first evaluate the structure, dynamics, and binding free energies of catecholate in differing binding poses and identify the effect of binding pose on free energy barriers of the rate-determining step (RDS). Finally, we provide our Conclusions.

## Methods

### Classical Molecular Dynamics

Classical MD simulations of COMT were carried out using the GPU-accelerated version[41, 42] of the AMBER 14 software package[41]. The starting structure was obtained from the COMT crystal structure (PDB ID: 3BWM[43]), which had bound SAM, dinitrocatecholate (DNC) inhibitor, and an Mg^2+^ cation. Three resolved water molecules in the X-ray crystal structure that were buried in the active site were kept in the simulations while external waters were replaced during protein solvation. For catecholate structures, nitro groups were removed from the DNC structure. The protein was described by the AMBER ff12SB[44] force field, which is derived from the ff99SB[45] force field with updates to backbone torsional parameters. For SAM and catecholate or DNC substrates, we employ the generalized AMBER force field (GAFF)[46] with partial charges assigned from restrained electrostatic potential (RESP) charges[47] obtained with GAMESS-US[48] at the Hartree-Fock level using a 6-31G*[49] basis set, as implemented by the R.E.D.S. web server[50–52]. Thoroughly tested parameters for Mg^2+^ were obtained from Ref. [53] (S1 Table and S1 Fig). The charge of protein residues was assigned with the H++ webserver[54–57] assuming a pH of 7.0 to yield -8 (-6) for the apoprotein (holoprotein). H++ assigns the protonation state of neutral histidine residues based on van der Waals’ contacts and hydrogen bonding distances, which results in His12, His142, and His192 being protonated at the δ position and His16, His57, and His182 being protonated at the ε position. The protein was solvated with a 10 Å buffer of TIP3P[58] water on all sides (a total average size of around 62x69x71 Å during the NPT production runs) and neutralized with 6 Na^+^ ions.

Several multistage equilibration protocols were employed, which differed only by the extent and nature of restraints that enforced the crystal structure active site coordination (S1 Text, S2–S6 Figs and S2 Table). These protocols included restrained and/or unrestrained minimizations, a quick NVT heating stage, NPT equilibration, and NPT production dynamics at T = 300 K and p = 1 bar. We used a Langevin dynamics thermostat with a collision frequency of 1.0 ps^-1^ and a random seed to avoid synchronization artifacts. For constant pressure dynamics, a Berendsen barostat with a pressure relaxation time of 1 ps was used. The SHAKE algorithm[59] was applied to fix all bonds involving hydrogen, permitting a 2 fs timestep to be used for all MD. For the long-range electrostatics, the particle mesh Ewald method was used with a 10-Å electrostatic cutoff.

### Free energy surfaces

**Fig 1:**
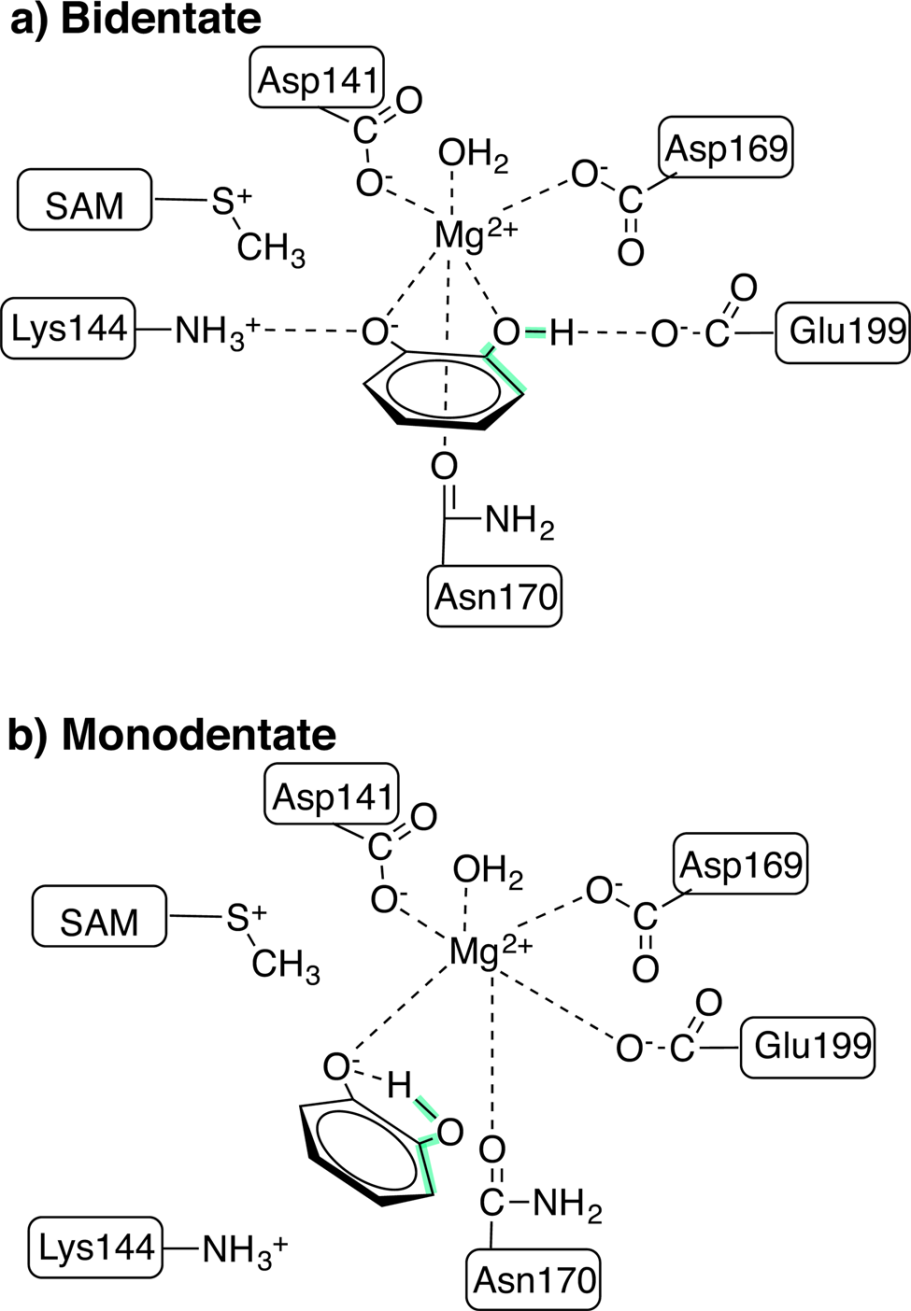
Model of key active site residues in COMT. a) Bidentate and b) monodentate configurations of COMT, including Mg^2+^ and its coordinating residues, catecholate and its interactions with Lys144 and Glu199, and the SAM substrate. The C-C-O-H dihedral angle is highlighted in green.

Two-dimensional MD free energy surfaces (FES) were obtained by applying the weighted-histogram analysis method (WHAM)[60, 61] using the Grossfield lab software package[62] to unbias distributions obtained with umbrella sampling[63]. Equally spaced windows were obtained at 0.1 Å spacing over the range of 1.8–4.5 Å in the Mg^2+^-OH bond distance and 5° spacing over the 0–180° range in the C-C-O-H dihedral (see Fig 1). Force constants were 500 kcal/(mol^.^Å^2^) for the bond distance restraint and 200 kcal/(mol^.^rad^2^) for the dihedral restraint. Each window was equilibrated for 100 ps before 200 ps of production dynamics was carried out over which sampled distances and dihedrals were collected. During the WHAM fitting, the iterative solution of the free energy weights was converged with a 1x10^-8^ threshold, and the final FES was described by 76 C-C-O-H dihedral bins of 2.5° width and 104 Mg^2+^-OH distance bins of 0.025 Å width.

### Quantum Mechanical FESs

Hybrid semi-empirical quantum-mechanical/molecular mechanics (SQM/MM) dynamics employed the internal AMBER[41, 42] SQM routines. These SQM/MM calculations include electronic embedding of SQM atoms and hydrogen capping of all cleaved covalent bonds that span the SQM/MM boundary. For the SQM approach, both AM1[64] with explicit treatment of *d* electrons and PM6[65] were employed. These dynamics were carried out with spherical boundary conditions in the largest sphere afforded by the MD box from which they were extracted with no electrostatic cutoff, a 0.5 fs timestep, and constant temperature (T = 300 K) enforced by a Langevin thermostat as in the MD runs. No atoms were held fixed, but a 1.5 kcal/(mol^.^Å^2^) restraining potential kept water molecules from evaporating from the sphere. Initial configurations across the methyl transfer coordinate were obtained from a quick steered molecular dynamics (SMD) run. Snapshots from the SMD runs were extracted for each of the ten windows in umbrella sampling for 10 ps equilibration and 20 ps production SQM/MM.

The reaction coordinate in both SMD and umbrella sampling was defined as an antisymmetric linear combination of distances (LCOD) between the S-C and C-O bonds, which break and form, respectively, during methyl transfer. Variable force constants ranging from 10 kcal/(mol^.^Å^2^) in low energy regions to 240 kcal/(mol^.^Å^2^) in high energy regions were employed to minimize the number of windows required while maximizing overlap over the -1.5 to 2.2 Å LCOD range (details of force constants, window targets, and window widths are provided in S3 Table). WHAM[60, 61] software[62] was used to reconstruct one-dimensional free energy curves with 0.02 Å bin widths. In order to validate PM6 for FESs, additional geometry optimizations were carried out both at the hybrid density functional theory (DFT, B3LYP[66–68]) and PM6[65] levels of theory (S2 Text).

### Energy Decomposition Analysis

Binding free energy calculations employed the AMBER MMPBSA.py[69] utility, which follows protocols outlined in Ref. [70]. New 5 ns MD trajectories were generated starting from representative bidentate, monodentate, and local max geometries for a single trajectory protocol. The local max geometry represents a high-energy point on the transition from bidentate to monodentate (see Results and Discussion) and was sampled with distance restraints, as described above. For this method, implicit solvent calculations within the Poisson-Boltzmann (PB)[71] or Generalized Born (GB)[72] approximations are carried out on snapshots obtained from MD both with a noncovalent ligand present and rigidly removed. In our simulations, the rigid binding free energies were averaged from configurations extracted every 8 ps for a total of 625 snapshots.

MMPBSA total binding free energies of Mg^2+^ and catecholate as well as the individual residue contribution to the binding energy of catecholate were obtained. Full energy decomposition analysis with MMPBSA is computationally intensive, and pairwise residue interactions were computed instead with MMGBSA using the "OBC1" model[73], motivated by recent benchmarks[74]. In both MMPBSA and MMGBSA cases, the internal dielectric was set to 1, and the salt concentration was set to 0.1 M. Entropic contributions to binding computed within the quasi-harmonic approximation were not found to vary across points being compared and were therefore neglected. More description of contributions to the MMPBSA binding free energies is provided in S3 Text.

## Results and Discussion

### Structure and Dynamics in the Active Site

At least ten experimental crystal structures[15, 43, 75–79] of COMT have been solved with bound inhibitors ranging from dinitro to coumarine in nature along with SAM and Mg^2+^ in the active site at resolutions ranging from 1.3 to 2.4 Å. On average, the bidentate substrate analogue in these structures has two Mg^2+^-O bond distances averaging around 2.16 Å that are comparable to the 2.12 Å average distance for the remaining species in the active site (Asp141, Asp169, Asn170, and H_2_O) that coordinate Mg^2+^. It has been proposed[14, 15] that the inhibitor molecule was bound in a monoanionic form. Based on expected pK_a_s, it is believed that Glu199 forms a hydrogen bond with the hydroxyl of one catechol, and the other oxygen proximal to the methyl group of SAM is deprotonated by Lys144 (Fig 1A).

Some simulations[27, 32, 80] have identified alternate active site configurations for catecholate in which a monodentate (m) structure with an intramolecular catecholate hydrogen bond is formed. The intramolecular hydrogen bond is known to be stable in gas phase structures of catecholate[81]. The newly available sixth coordination site on Mg^2+^ is either filled by monodentate coordination to Glu199[80] or bidentate coordination with Asp141[27, 32] (Fig 1B). The bidentate Asp141 configuration is likely an artifact of force field parameter choice, as bidentate coordination of Mg^2+^ is exceedingly rare under physiological conditions[82]. An alternate m configuration (m-alt)[33] has been proposed in which the neutral hydroxyl oxygen of catecholate weakly binds Mg^2+^ with a > 2.5 Å Mg^2+^-O(H) bond and the sixth Mg^2+^ coordination site is instead occupied by Glu199. This binding orientation necessitates rearrangement in the active site or proton transfer along a path that crosses very close to Mg^2+^, and the weak catecholate-Mg^2+^ interaction is unlikely to be sustained during the relatively slow catalytic cycle of COMT (turnover frequency of 12–24 min^-1^ [21, 22]). When we sampled neutral catechol or a species in which only the neutral hydroxyl oxygen of catecholate was directly coordinated to Mg^2+^, we observed extremely short (i.e., sub-ns) lifetimes for that species. In an attempt to stabilize m-alt, we also carried out restrained MD in the configuration proposed in Ref. [33] for 50 ns before release and were able to stabilize this configuration for up to 50 ns before it rearranged to the standard m configuration.

**Fig 2:**
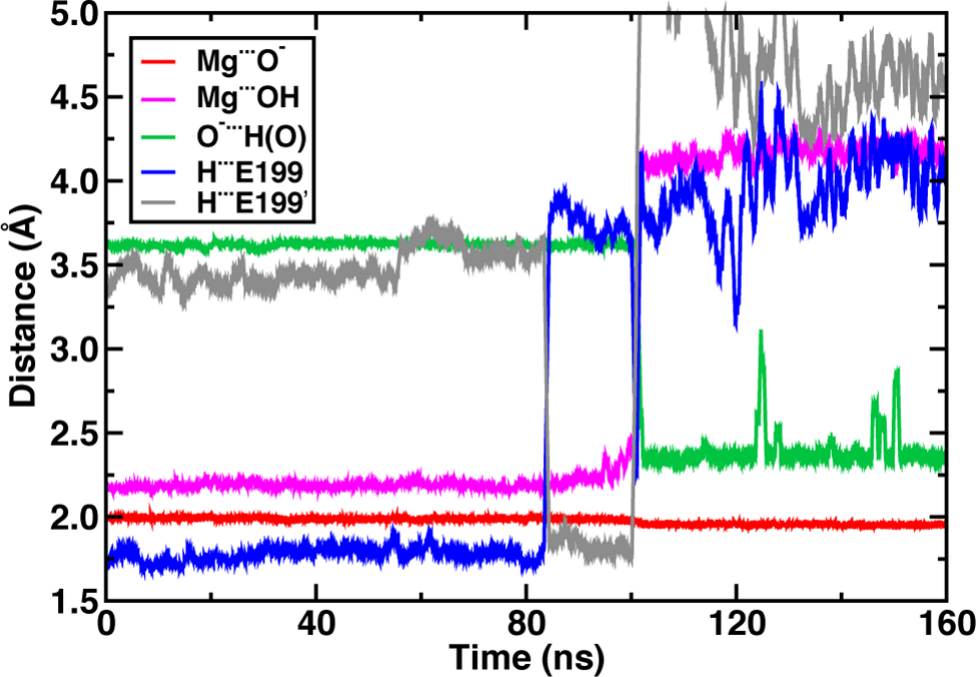
Active site bond distances from a 160 ns molecular dynamics trajectory. Distances include Mg^2+^ coordination with catecholate anion (red line) and hydroxyl oxygen atoms (magenta line); catecholate intramolecular hydrogen bond distance (green line); and catecholate hydrogen bond distance to Glu199 carboxylate oxygen atoms (blue and gray lines).

As with previous work[27, 32, 80], we also observe rapid bidentate (b) to monodentate (m) rearrangement (Fig 2) of catecholate-Mg^2+^ coordination (Fig 1), depending upon the equilibration protocol employed (S2–S6 Figs and S1 Text). The most rapid rearrangement is observed in protocols in which no restraints are applied to the protein or ligand environment during equilibration. Use of harmonic restraints on Mg^2+^-O distances during NVT heating and NPT equilibration leads to b structures that are stabilized for at least 80 ns before rearrangement to a m structure (Fig 2, see S1 Text for a full 350 ns bidentate run when dihedral restraints are employed). The long lifetime of the b species departs from unrestrained equilibration protocols in earlier[27, 32, 80] short timescale MD and our own MD. Analysis of bond distance changes reveals that the dynamical rearrangement is induced by the destabilization of the catecholate hydroxyl-Glu199 hydrogen bond via Glu199 sidechain rotation. This fluctuation encourages the catecholate hydroxyl to reorient, forming an intramolecular hydrogen bond and monodentate coordination to Mg^2+^. Shortly thereafter, Glu199 is observed to coordinate directly to Mg^2+^. In all simulations, no reverse transition from m to b coordination is observed on the 100-ns timescale.

**Fig 3:**
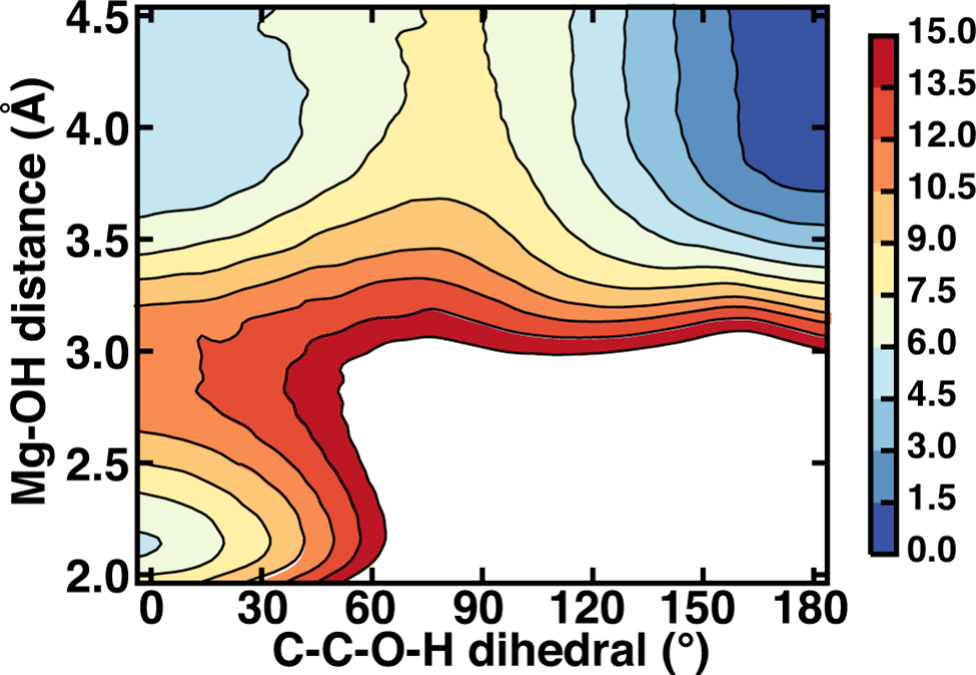
Catecholate-Mg^2+^ coordination FES in COMT with respect to C-C-O-H dihedral and Mg-OH distance. Color bar at right shown in kcal/mol with high free energy regions in excess of 15 kcal/mol shown in white.

The electrostatic driving force for the catecholate rearrangement during classical MD simulations is clear: oxygen partial charges on catecholate are weaker than those on Glu199 (S7 Fig). However, a key question is whether the overall free energy of the m configuration is substantially lower than b since the lack of m-to-b rearrangement during MD suggests a higher barrier for the backward than the forward transformation and thus possibly a lower free energy for the m configuration. The two-dimensional (2D) free energy surface (FES) spanning Mg^2+^-OH distances and C-C-O-H dihedral values confirms observations from MD (Fig 3). The minimum free energy path involves Mg-OH bond elongation followed by formation of the intramolecular hydrogen bond (C-C-O-H dihedral of 180°, see Fig 1 for dihedral location on catecholate). The free energy barrier for the Mg^2+^-OH bond elongation step of around 6.5 kcal/mol is consistent with observations of a long-lived b structure during MD simulations.

A second free energy minimum that is equienergetic with the bidentate structure corresponds to elongated Mg^2+^-OH distances in a monodentate configuration without the intramolecular catecholate hydrogen bond. Here, a hydrogen bond with Glu199 stabilizes the hydroxyl of monodentate catecholate instead (see Fig 1). Rearrangement to a m structure with an intramolecular hydrogen bond has a low free energy barrier of around 3 kcal/mol. Overall, the m-catecholate (m-CAT) with an intramolecular hydrogen bond is stabilized by around 4 kcal/mol with respect to the other two free energy basins. The m-alt configuration employed in Ref. [33], on the other hand, was found to be 11 kcal/mol higher in energy than a bidentate reference, and interconversion to m-alt is prohibitive with a free energy barrier of 24 kcal/mol (S8 Fig).

**Fig 4:**
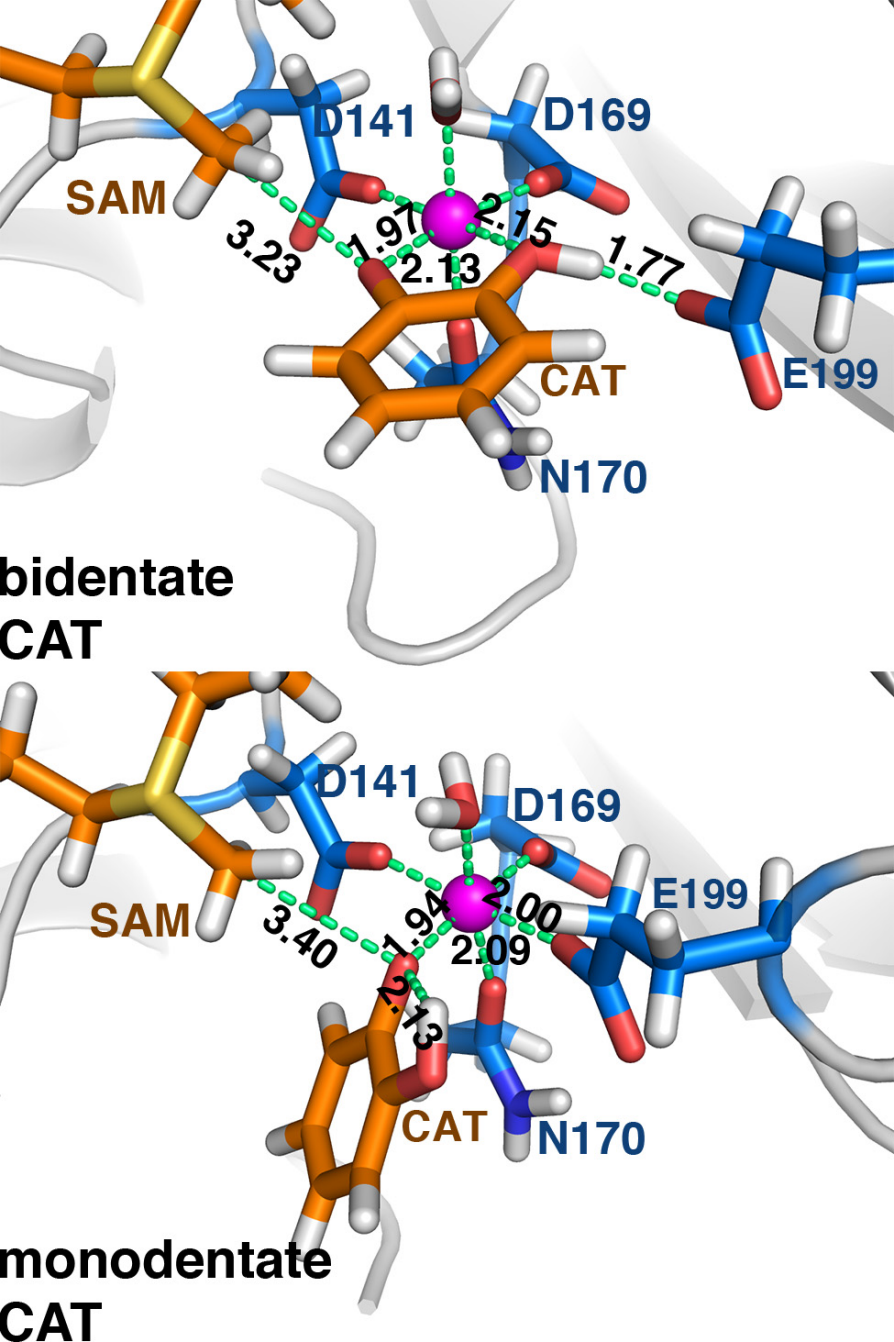
**Representative bidentate (top) and monodentate (bottom) catecholate (CAT) substrate configurations at the COMT active site.** Substrates are shown in orange, protein residues in blue, and key distances are shown (in Å), except for D141-Mg^2+^, D169-Mg^2+^, and Mg^2+^-H_2_O, which are unchanged.

During MD sampling of m-CAT, we observe occasional interconversion between the intramolecular H-bond monodentate (C-C-O-H dihedral = 180°) and the extended hydroxyl case (C-C-O-H dihedral = 0°) consistent with the features of the free energy surface. Although no rearrangement back to b-CAT is observed, the barrier for conversion from the m-CAT, intramolecular H-bond catecholate to b-CAT is predicted to be 10.5 kcal/mol, corresponding to an interconversion frequency of 16 μs^-1^. This exchange frequency is within an order of magnitude of experimental 0.67 μs^-1^ exchange rates at room temperature (1.5 μs^-1^ at 37°C) of neutral ligands such as H_2_O around Mg^2+^[83] and should occur multiple times between the slower RDS methyl transfer steps. Representative structures from b-CAT and m-CAT free energy basins reveal key differences in orientations (Fig 4). Since X-ray structures are usually solved in the presence of a dinitrocatecholate (DNC) inhibitor, we also carried out MD simulation with DNC bound and confirmed that the b-to-m rearrangement was comparable to CAT (S9 Fig).

In both CAT and DNC MD simulations, a short hydrogen-Glu199 oxygen distance of around 1.7 Å in the b-CAT configuration is replaced by a 2.1 Å intramolecular hydrogen bond in the m-CAT case (S4 Table). Whereas the CAT oxygen anion coordination distance to Mg^2+^ is always predicted to be shorter than crystal structure values (1.94 Å in m-CAT, 1.97 Å in b-CAT), the longer DNC distances (2.08–2.16 Å, S9 Fig) are consistent with experimental crystal structure distances. Overall distances between both substrate and inhibitor are comparable, with short 1.90–1.94 Å distances for Mg^2+^ coordination to carboxylates of Asp141 and Asp169 and longer 2.02–2.13 Å bond distances with Asn170 and the axial water ligand. The neutral catechol hydroxyl coordination distance to Mg^2+^ is consistently longer by 0.15–0.25 Å in both CAT and DNC with respect to the compensating interaction that is formed with the Glu199 carboxylate, consistent with analysis of the charges of each residue in the active site (S7 Fig and S4 Table). Interestingly, distances of the transferring methyl carbon on SAM to the nearest oxygen on catechol vary in the b and m MD configurations. Crystal structures have unusually short SAM C–CAT O distances (average: 2.65 Å, range: 2.45–2.81 Å), and it has been hypothesized that unusually short reactant distances may be a source of catalytic rate enhancement[25].

**Fig 5:**
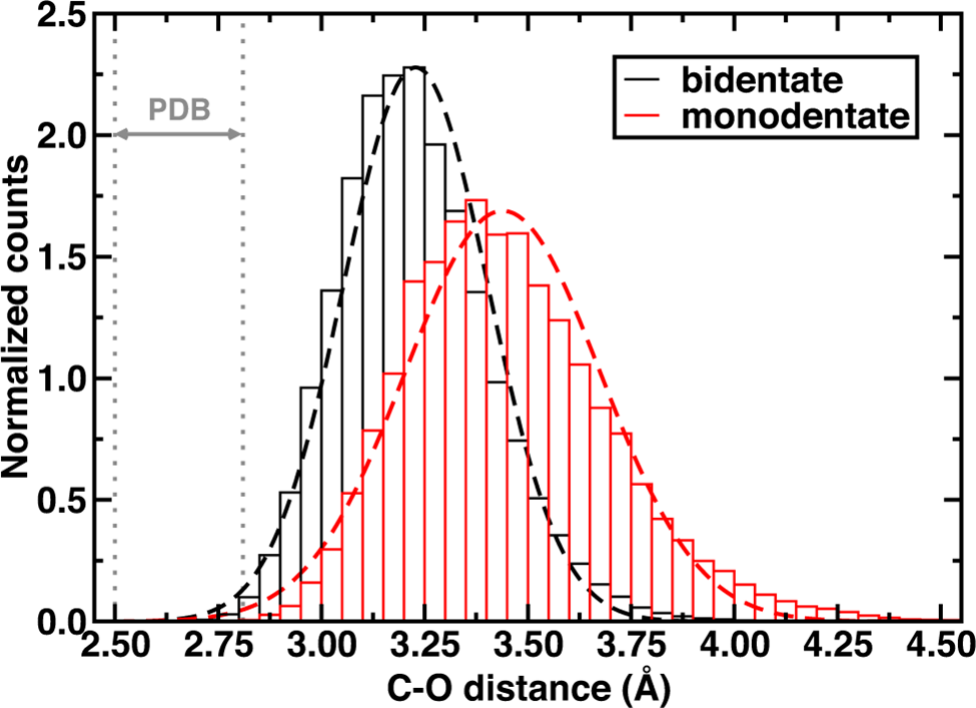
Histograms of C(SAM)-O^-^(catecholate) distances for bidentate (black lines) and monodentate (red lines) catecholate-Mg^2+^ coordination in COMT. Dashed lines are the best-fit normal distributions. The range of C-O distances in X-ray crystal structures of COMT is indicated by two gray vertical dotted lines.

The GAFF repulsive van der Waals terms for SAM and CAT prevent extensive sampling of C-O distances observed in X-ray structures. This repulsion is slightly balanced by weak electrostatic attraction between methyl hydrogen atoms (*q* = +0.12 *e*-) and the anionic oxygen of the substrate (*q* = -0.55–0.80 *e*-). Weaker partial charges on the DNC oxygen atoms lengthen the C-O distances even further (S4 Table). There are still distinguishable differences in sampled SAM-CAT distances for the two CAT m and b binding orientations (Fig 5). The b-CAT configuration samples distances shorter than 3.2 Å (or 2.8 Å) between a methyl C and O^-^ 42% (4%) of the time, whereas m-CAT only samples those distances for 11% (0.3%) of the trajectory. Both the b-CAT and m-CAT C-O distances may be fit to normal distributions with means of 3.22 Å and 3.44 Å, standard deviations of 0.175 Å and 0.236 Å, and minimum distances of 2.65 Å and 2.75 Å, respectively. Semi-empirical AM1 QM/MM geometry optimizations have indicated a C-O distance of 2.92 Å in an alternate, weakly-coordinating monodentate configuration (m-alt)[33], but the closest comparison to our room temperature MD result from the same level of theory instead indicated a 3.25–3.50 Å[84] preferred C-O distance in that orientation. Classical MD on the m-alt structure reveals a comparable C-O distribution for the Mg^2+^-coordinated hydroxyl oxygen (3.44 Å) and a broader and longer C-O distance for the free anionic oxygen (centered about 4.0 Å). This longer distance indicates that the anionic oxygen is seldom available to form a catalytically competent geometry and thus this configuration is not considered further (S10 Fig). We will revisit QM/MM equilibrium bond distances from QM/MM MD in the next section, where the possible effect of the different starting structures on the methyl transfer RDS will now be considered in more detail.

### Substrate-position-dependent Free Energy Barriers

We now investigate how substrate-positioning differences affect predicted free energy barriers for the rate-determining methyl transfer step. Although a number of computational studies have made a range of predictions for the COMT methyl transfer free energy barrier[27, 32, 33, 37, 85] (3–30 kcal/mol), a systematic comparison of methyl transfer barriers between the distinct bidentate and monodentate substrate configurations has not been carried out.

Recent work by some of us[24] has indicated that properties of COMT converge only with inclusion of 100s of atoms in the QM region in QM/MM calculations. It appears that accurate free energy calculations should be carried out with charge transfer (CT) permitted between the substrates, Mg^2+^, and the protein environment. In order to enable sufficient sampling but maintain a sufficient description of the substrate electronic environment, we turn to computationally efficient semi-empirical methods and validate them with hybrid DFT. Semi-empirical models from Stewart and coworkers have been demonstrated[86] to describe Mg^2+^ hydration free energies well. The central importance of Mg^2+^ in this protein motivated our selection of the PM6 semi-empirical Hamiltonian as well as its availability in the AMBER code. Three SQM region sizes were considered: catecholate and SAM substrates only (S region), substrates with Mg^2+^ cation (SMg), and the substrates, Mg^2+^, and Mg^2+^ ligands (Asp141, Asp169, Asn170, Glu199, and water) as well as Lys144 (SMgL). Recall that in b-CAT, Glu199 hydrogen bonds with CAT whereas in m-CAT Glu199 directly coordinates Mg^2+^. Lys144 is included in order to allow for proton transfer or hydrogen bonding with CAT. The largest model selected here (SMgL) contains most of the critical residues used in previous large-scale QM/MM models[24].

**Fig 6:**
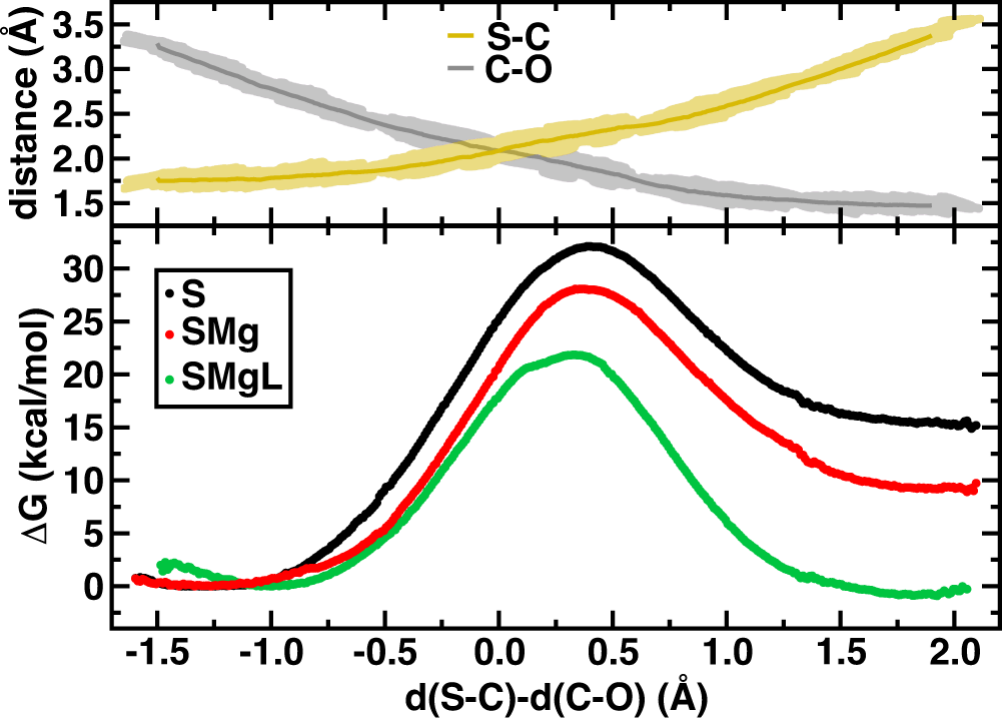
Bidentate methyl transfer free energy curves. (bottom) Methyl transfer free energy curves from PM6/MM (in kcal/mol) for three models of increasing size: catecholate and SAM substrate-only (S, black circles), with Mg^2+^ (SMg, red circles), and with Mg^2+^ coordination ligands (SMgL, green circles) plotted against the difference of S-C and C-O bond distances. (top) Absolute, average and range of S-C (yellow line) and C-O (gray line) distances (in Å).

Umbrella sampling was carried out to obtain the free energy for methyl transfer at the PM6/MM level of theory with an LCOD coordinate that was the difference (Δ) between the donor (S-C) bond and the acceptor (C-O) bond (sampled values illustrated in top panel of Fig 6). We observe a significant decrease in the methyl transfer free energy barrier as the SQM region size is increased (Fig 6), with a 6.3 kcal/mol drop from SMg to SMgL SQM regions. Only with the full SMgL region do we recover the prediction of an exothermic methyl transfer step, whereas the other two regions correspond to strongly endothermic reactions (see Fig 6 and S5 Table). Overall prediction of a 21.8 kcal/mol free energy barrier for b-CAT is in good agreement with the experimental value of 18.1–19.2 kcal/mol[21, 22]. Continued QM region enlargement, which is currently computationally prohibitive, could improve agreement further.

In addition to the methyl transfer barrier, our sampling of the difference (Δ) between S-C and C-O distances provides information about the FES of the Michaelis complex. For all SQM regions the free energy surface is relatively flat over the range of Δ = -1.5 to -0.75 Å, but the free energy minimum shifts significantly from S to SMgL. The equilibrium C-O distance in b-CAT S SQM/MM is 3.12 Å, around the same as observed in MD, but this value decreases to 2.98 and 2.72 Å for SMg and SMgL SQM/MM, respectively (Fig 6, Table 1 and S6 Table). The CT-mediated minimum free energy C-O non-bonded distance in the SMgL SQM/MM Michaelis complex is consistent with the range of distances observed in crystal structures (2.45–2.81 Å). We note absolute bond distances are subject to the errors inherent in PM6 at around 0.03–0.10 Å[65], but trends in distances with QM-region likely benefit from cancellation of errors.

**Table 1:** Geometries of the enzyme-substrate (ES) complex and transition state (TS) for m-CAT and b-CAT configurations for the SMgL PM6 SQM region.

	b-CAT ES	m-CAT ES	b-CAT TS	m-CAT TS
S-C (Å)	1.76	1.76	2.21	2.19
C-O (Å)	2.72	3.02	1.91	1.90
Δ (Å)	-0.96	-1.26	0.32	0.29
∠S-C-O (°)	158.7	162.7	173.2	173.3

The CT-mediated C-O distance shortening appears strongly correlated to the reduction in free energy barriers. In fact, the three SQM regions can be fit (R^2^ = 0.99) to the expression:

$$\Delta G^{‡}=25.5\times d(\text{C-O})-47.8,$$ (1)

where *d*(C-O) is the C-O distance in Å of the Michaelis complex and the units are in kcal/mol (S5 Table). This observation suggests that within QM treatments of COMT, shorter distances are correlated to greater recovery of electronic contributions to enzymatic rate enhancement. However, we have not yet considered the effect the QM region size has on the highest energy point, which we refer to approximately as the transition state (TS) of the reaction coordinate.

Examining the b-CAT methyl transfer TS geometries (see Table 1 and S6 Table) reveals some S-C distance shortening as the SQM region is increased (from 2.28 to 2.21 Å for S to SMgL) but no significant difference in C-O distances (1.88 Å to 1.91 Å). However, this reduction in S-C distance pushes the transition state earlier on the Δ coordinate from 0.40 to 0.32 Å. Our SQM/MM results are consistent with or correspond to a slightly tighter transition state compared to distances in previous semi-local DFT/MM studies of 2.24 Å and 2.07 Å for S-C and C-O, respectively[85]. Differences in the S-C-O angle reveal some region dependence with the SMgL SQM/MM calculations showing the closest correspondence between the Michaelis complex and the TS of a little under 15°. Therefore, in the SMgL simulations, the effective barrier height reduction is arising because the TS and MC resemble each other more closely than in the smallest S SQM/MM calculations.

We now compute the region dependence of the free energy barriers for methyl transfer to m-CAT (S11 Fig, S5 and S6 Tables). We again observe a decrease in the free energy barrier as the SQM region is increased along with a shortening of the C-O distance in the Michaelis complex. Here, the S SQM/MM C-O distance of 3.38 Å is comparable to the classical MD m-CAT free energy minimum, and it shortens to 3.02 Å in the SMgL SQM/MM simulation. The distance shortening with SQM region increase is somewhat smaller in m-CAT than b-CAT (0.36 versus 0.40 Å), consistent with reduced CT and interaction with Mg^2+^ for m-CAT vs. b-CAT. Although the distance decrease is smaller, the free energy barrier reduction from S to SMgL SQM/MM is only slightly reduced (10.3 kcal/mol for bidentate to 8.7 kcal/mol for monodentate, see Table 2 and S5 Table). The resulting correlation between the free energy barrier and Michaelis complex is of similar magnitude to that for b-CAT, albeit with a reduced correlation coefficient of 0.85.

**Table 2:** Free energy barriers (ΔG^‡^) and reaction free energies (ΔG_Rxn_) for methyl transfer from the monodentate and bidentate catecholate configurations with the SMgL SQM region in SQM/MM calculations.

	ΔG^‡^ (kcal/mol)	ΔG_Rxn_ (kcal/mol)
bidentate	21.8	-0.9
monodentate	22.1	-2.4
expt.	18.1–19.2^a^	—

^a^Refs. [21, 22].

**Fig 7:**
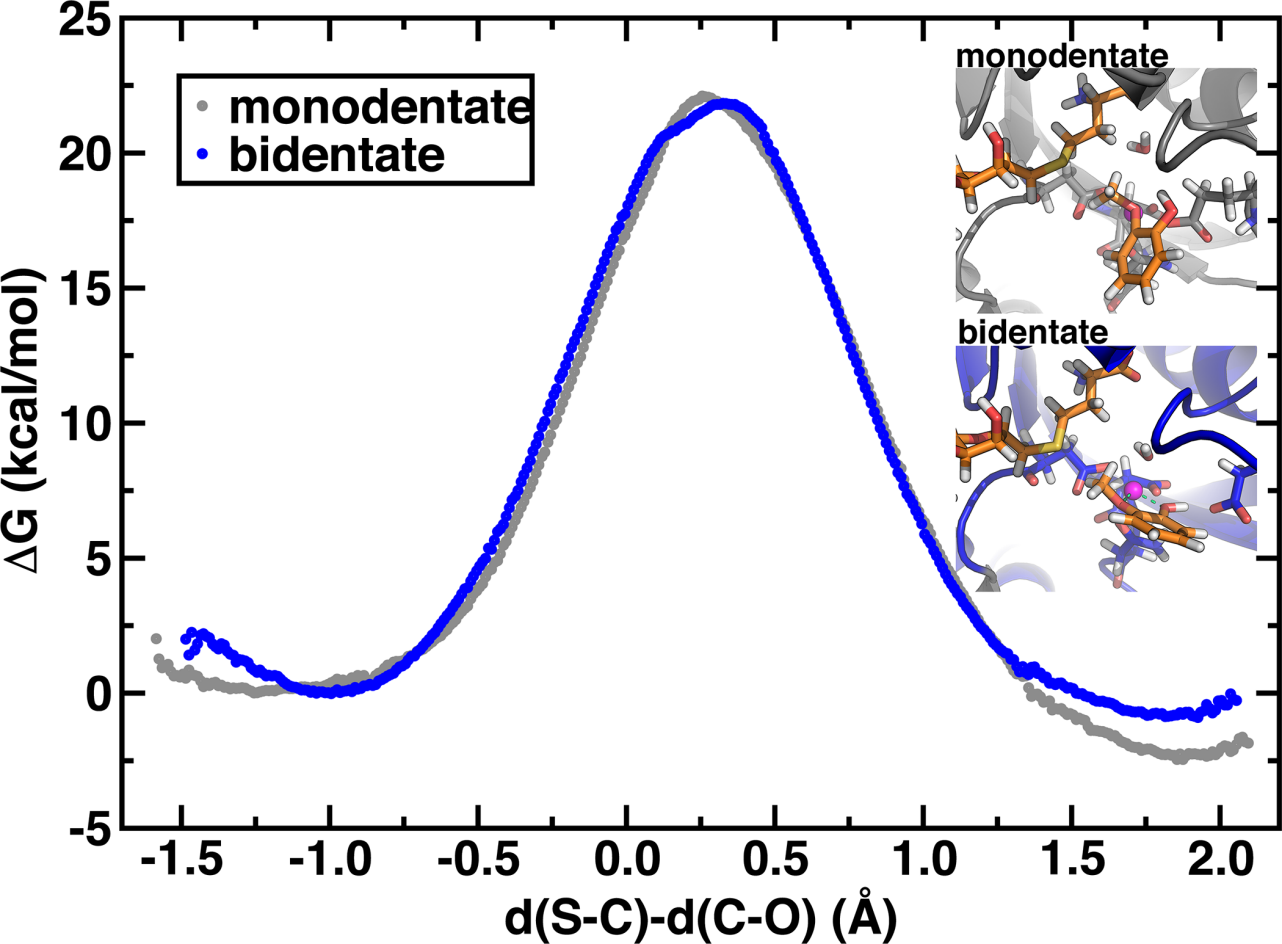
Bidentate and monodentate methyl transfer free energy curves. Methyl transfer free energy curves (in kcal/mol) for the SMgL model for bidentate (blue circles) and monodentate (gray circles) catecholate-Mg^2+^ coordination plotted against the difference in S-C and C-O distances (in Å). Representative product geometries are shown in the inset.

Overall comparison of the bidentate and monodentate methyl transfer for the largest SQM region considered reveals nearly identical methyl transfer energetics (Fig 7 and Table 2). The S-C and C-O distances of the m and b TSes are also nearly identical (see Table 1). It has been suggested[34] that m-CAT orientation, especially with the catecholate oxygen anion uncoordinated to Mg^2+^, might be necessary in order to make the catecholate oxygen anion a more suitable nucleophile for the S_N_2 methyl transfer and that the b-CAT form in X-ray structures is an inactive state of the enzyme. The nearly identical methyl transfer barriers of 21.8 and 22.1 kcal/mol for b-CAT and m-CAT, which are both slight overestimates of the experimental range[21, 22] (18.1–19.2 kcal/mol), however, suggest the oxygen atoms of both coordination geometries are equally suitable nucleophiles. One could draw a different conclusion that the m-CAT structure was preferred if one used the CT-restrictive S SQM region results. The m-CAT reaction is predicted to be slightly more exothermic, as a consequence of a smaller effect on Mg^2+^ stabilization by CAT when the methylated m-CAT product is formed (see inset of Fig 7). [87]A key distinction for future study is that the b-CAT Michaelis complex C-O distance is dramatically shorter by nearly 0.4 Å, which may enhance reaction probability by impacting recrossing from that configuration and would need to be addressed by considering a generalized transmission coefficient.[87]

We also considered the extent to which the choice of the PM6 semi-empirical method has impacted predictions of the methyl transfer free energy barriers. Earlier[33] AM1/MM calculations had used an MP2-derived correction in order to add > 10 kcal/mol to the semi-empirical free energy barriers. However, two major differences in those calculations from the current work were the exclusion of Mg^2+^ from the SQM region and the use of a reactant reference in which Mg^2+^ was only weakly coordinated by the neutral hydroxyl oxygen of catechol and average C-O distances ranged from 2.95[33] to 3.25[84] Å. We compared our AM1/d/MM and PM6/MM free energy barriers, where the AM1/d semi-empirical approach incorporates parameters for Mg^2+^ and found the AM1/d/MM results to produce comparable energy barriers (S12 Fig). In the previous work[33], a very weakly coordinating CAT reference was used as the reactant, likely causing the lower computed barrier for methyl transfer (see Fig 3). In addition to semi-empirical methods, we compared enthalpies obtained with hybrid DFT using the B3LYP functional with the 6–311++G* basis set. Using a number of techniques outlined in the Supporting Information including model calculations in gas phase and implicit solvent as well as clusters cut directly from the SQM/MM free energy curves, we confirmed consistency in barrier estimates between PM6 and hybrid DFT (S7 Table and S13 and S14 Figs). Overall, these results suggest that the electronic environment of COMT enforces a shortened C-O distance and lowers the free energy barrier for methyl transfer. Interconversion to a monodentate structure may occur on rapid timescales, but this structure likely is less, rather than more, reactive for methyl transfer.

### Differences in Substrate-Protein Interactions

Interactions between CAT, SAM, Mg^2+^, and protein appear distinct between b-CAT and m-CAT configurations. The electrostatic driving force for b-CAT to m-CAT rearrangement has been identified as the higher point charges for Glu199 than for the neutral catechol hydroxyl (S7 Fig). Nevertheless, electrostatic interactions are only part of free energy differences, and we now carry out binding free energy calculations and energy decomposition analysis (EDA) with MMPBSA in order to further identify differences in binding modes (S3 Text). The MMPBSA approach has been used before to analyze COMT inhibitor binding[27, 88, 89], but we leverage it for the first time to identify differences in b-CAT and m-CAT configurations.

We first computed total binding free energy components for CAT in a b-CAT, m-CAT, and local maximum (local max) configuration. The local max configuration corresponds to the highest free energy structure for a C-C-O-H dihedral around 0° on the 2D-FES (Fig 3) for the b-to-m transition. For these 3 orientations, we computed the CAT binding energy with a SAM- and Mg^2+^-containing receptor protein, consistent with experimental observations of the order of binding of substrates in COMT[12]. In addition to CAT binding, we considered the artificial case of treating Mg^2+^ as the ligand and the SAM-, CAT-containing protein as the receptor. Although not necessarily physical, this analysis enables us to answer the question of whether the overall stabilization of m-CAT is derived from stronger binding of Mg^2+^ or CAT to the protein.

**Fig 8:**
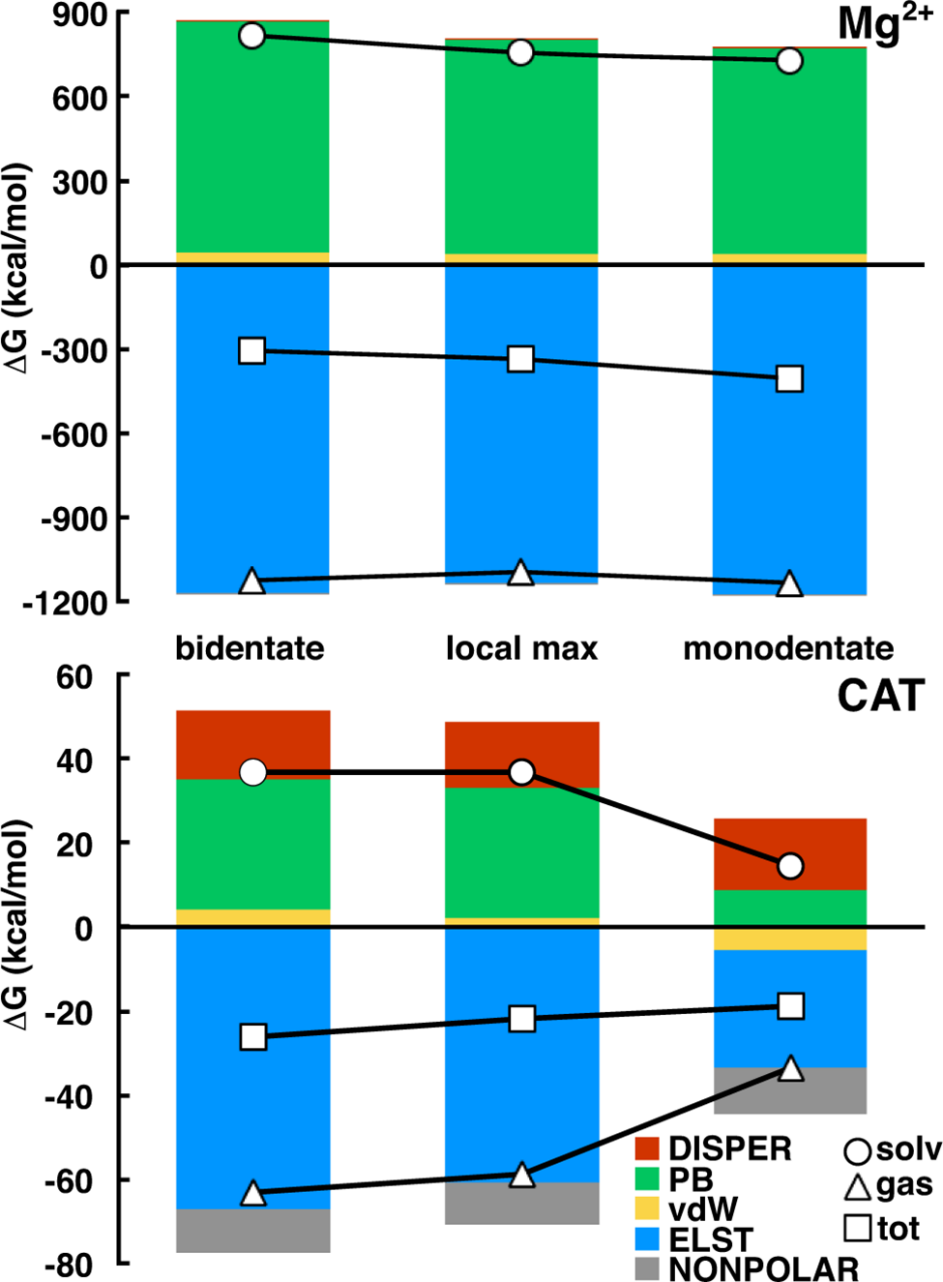
Binding free energy components. Binding components for Mg^2+^ (top) and catecholate (CAT, bottom) from MMPBSA for the bidentate (left), monodentate (right) and local max (center) configurations. Contributions of each bar for both graphs are shown in the legend (bottom right). Lines represent the total (squares), gas phase (triangles), and solvent (circles) contributions.

Total and component binding free energies for CAT and Mg^2+^ reveal that Mg^2+^ binding is enhanced and CAT binding weakened from the b-CAT to m-CAT configurations (Fig 8). In CAT, a 59% reduction in the electrostatic (elst) stabilization is observed from b-CAT to m-CAT, but this reduction is counteracted by other components to give an overall decrease in binding free energy of 28%. These components are the reduction in cost to desolvate m-CAT, indicated primarily through the less repulsive Poisson-Boltzmann (PB) polar solvation term as well as a shift from weakly repulsive van der Waals (vdW) substrate-protein interactions to weakly attractive ones. Other components of the binding free energy are relatively unchanged. The increase in binding free energy of Mg^2+^ (see Fig 8) in the b-to-m transition is primarily (92% of total free energy difference) in the reduction of PB solvation penalty. The direct difference in gas phase elst of binding provides a comparatively smaller (5%) contribution. Local max elst become unfavorable, which is a likely component of the free energy barrier observed earlier on the 2D-FES. These MMPBSA results suggest the lower free energy of m-CAT on the 2D-FES is due to a stabilization of Mg^2+^ and destabilization of CAT.

**Fig 9:**
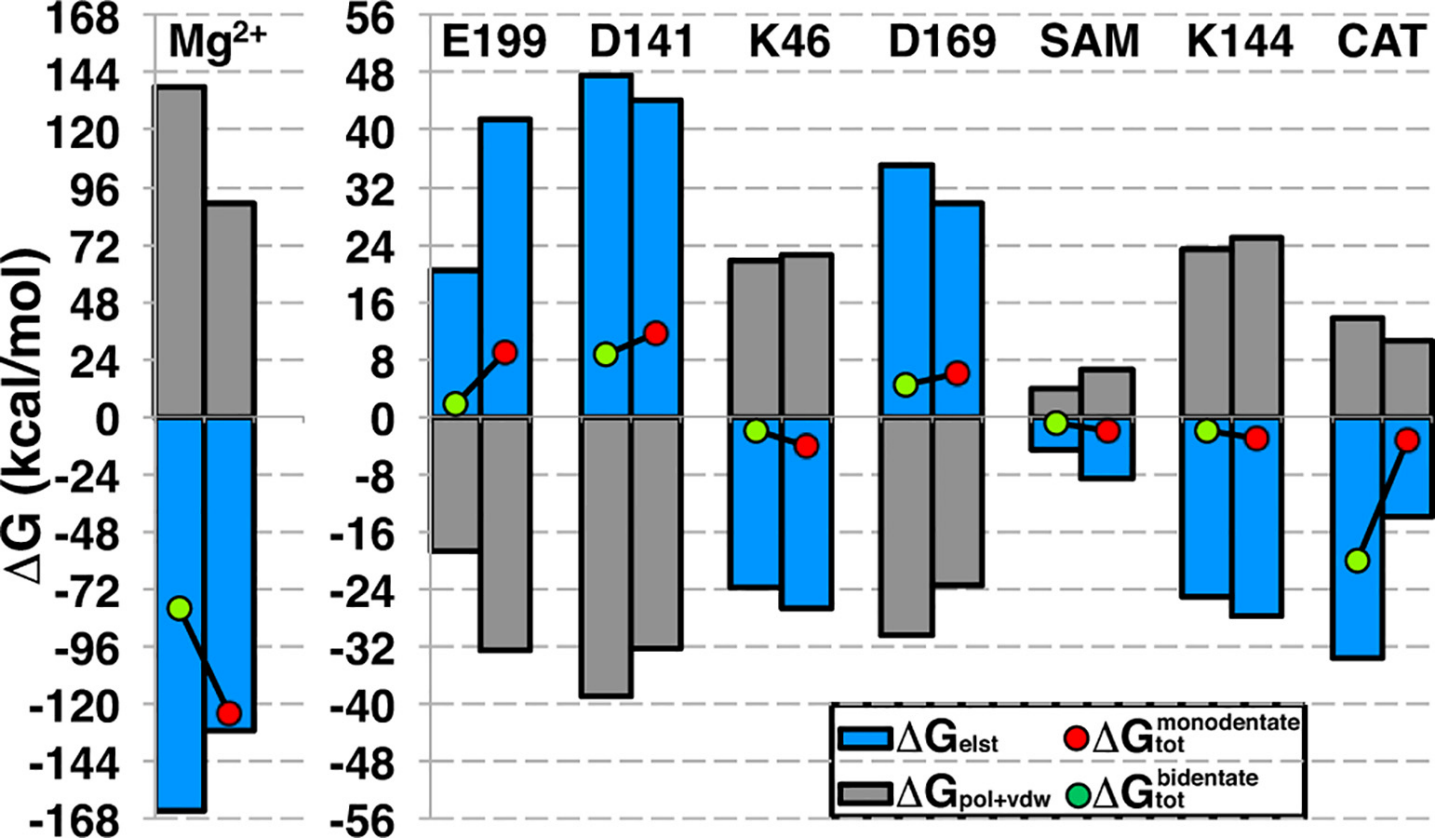
Residue contributions to catecholate binding. Residue contributions to binding in bidentate (green circle, left bar in clustered bars) and monodentate (red circle, right bar) binding free energy as well as elst (blue bar) and pol+vDW (gray bar) contributions.

Decomposition of individual residue contributions to the binding free energy also provides insight into how protein-CAT interactions shift between the b-CAT and m-CAT configurations. We identified the residues or substrates that had at least 1 kcal/mol binding free energy difference between b-CAT and m-CAT: Mg^2+^, SAM, CAT, Glu199, Asp141, Lys46, Asp169, and Lys144 (Fig 9). Glu199 had been previously identified (see Fig 4) as forming a hydrogen bond to b-CAT or alternately coordinating Mg^2+^ and repelling m-CAT, consistent with a 7 kcal/mol more repulsive contribution to m-CAT binding due to increasing elst binding penalty. The Mg^2+^-coordinating residues Asp141 and Asp169 are similarly more repulsive (3 and 2 kcal/mol, respectively), but, unlike Glu199, these differences are derived from the gas phase vdW and polar solvation (pol) contributions as a result of m-CAT's greater proximity to those residues. The increased electrostatic attraction of Lys144 and binding free energy contribution change (-1 kcal/mol) is due to increased hydrogen bonding between m-CAT and the NH_3_^+^ group of Lys144. Similarly, the protonated Lys46 is positioned on the neutral hydroxyl side of CAT. The b-to-m hydroxyl reorientation increases (-2 kcal/mol) favorable interactions between m-CAT OH and Lys46.

The remaining b-to-m shifts in residue contributions to binding energies are derived from SAM, CAT, and Mg^2+^ and recapitulate earlier observations of Mg^2+^ increasing binding and CAT decreasing binding (see Fig 8). This difference may be rationalized by an increase in solvent exposure of Mg^2+^ in the m-CAT configuration as well as a more stabilizing coordination sphere from Glu199 even in the absence of m-CAT (i.e. in the receptor alone). CAT gas phase electrostatic binding is greatly reduced (20 kcal/mol), although this effect is counterbalanced slightly by a reduction in the repulsive pol+vdW contributions for overall binding (17 kcal/mol). Changes in SAM contribution to binding are weakly attractive by -1 kcal/mol, likely due to reorientation of the CAT O^-^.

**Fig 10:**
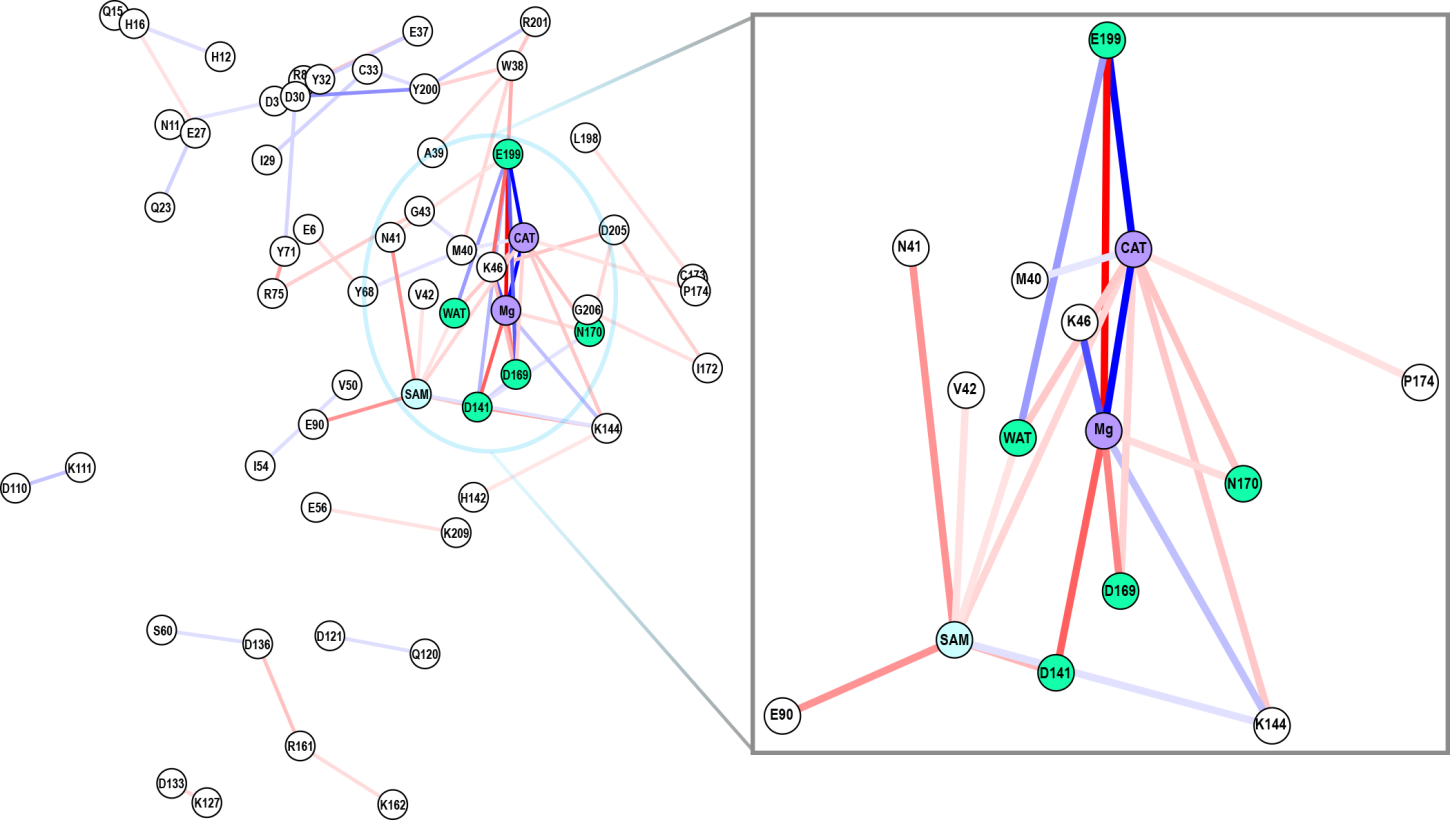
Network graph of interaction shifts. Graph (full left and inset right) of difference in total pairwise residue interaction energies between bidentate and monodentate catecholate binding (greater than 1 kcal/mol). Line color indicates strengthening (red) or weakening (blue) with saturation of colors at +/- 15 kcal/mol.

Finally, we consider the shift in residue-by-residue interactions for b-CAT and m-CAT using MMGBSA. We compare the absolute energies of the b-CAT and m-CAT complexes and identify a large number of residue pairs that contribute more than 1 kcal/mol difference in pairwise-residue total interaction energies. The map shown in Fig 10 is a 2D projection of the center of mass of each residue in the protein that has pairwise-residue interaction energy shifts indicated by a line connecting residues (red for increasing, blue for decreasing). Most of the strongest pairwise changes are adjacent to CAT in the active site and consistent with earlier EDA. From b-CAT to m-CAT, the CAT-Mg^2+^ interactions are weakened, Lys46 interactions increase with CAT and decrease with Mg^2+^, and Mg^2+^ interactions with nearly all other directly coordinating residues (Glu199, Asp169, Asp141, and Asn170) are strengthened. Other strong b-to-m shifts are strengthened binding of SAM to the protein via a SAM carboxylate-Asn41 sidechain interaction and hydrogen bonding to the Glu90 sidechain.

Subtler shifts in pairwise interactions highlight key residues that have been identified in earlier work but also suggest new interactions that may warrant further study. In m-CAT, the "gatekeeper" residues[12] Trp38 and Pro174 have strengthened interactions with Glu199 and CAT, respectively. Tyr68 interactions with Glu6 strengthen while interactions with Met40 weaken, suggesting a slight movement of Glu6 and Tyr68 away from the active site in the monodentate structure. COMT Tyr68Ala mutants have been shown experimentally and computationally[22, 24] to be less catalytically competent, strongly implicating Tyr68 as a key residue to mediate methyl transfer. Although Tyr200 has been identified as a component of the substrate binding pocket[25], one strong pairwise interaction shift involving residues not highlighted in previous work is the Asp30-Tyr200 pairwise interaction. The change in sidechain hydrogen bonding between the solvent- and active-site-facing loop Tyr200 and α-helix Asp30 disrupts the loop orientation. This observation is consistent with experiments that have identified significant loop movements are occur in COMT when comparing an ensemble of structures ranging from apo to holo forms[90, 91].

Another important cluster of interaction shifts is the strengthening of Arg161 pairwise interactions with Asp136 and Lys162 along with Asp136 and Ser60 pairwise interaction weakening from b to m configurations. These residues are maximally distant from the active site, adjacent to the well-studied[22, 24, 29, 30, 43, 92] Val108Met polymorph site and could play a similar role in altering the solvent accessibility of the active site and stability of the protein as was suggested for Val108Met. More proximally, the α-helical residues Tyr71 and Arg75 have a strengthened interaction adjacent to a number of active site residues. These results motivate future extensions to use this energy decomposition analysis to guide computational mutagenesis.

## Conclusions

We have used classical and semi-empirical quantum mechanical methods to investigate multiple substrate binding modes observed in computation and experiment and how they influence mechanistic predictions in COMT. At the classical level, a monodentate CAT structure in which only a single oxygen anion is coordinated to Mg^2+^ is preferred by 4 kcal/mol over the experimental structure in which both CAT oxygen atoms are coordinated to Mg^2+^. However, the free energy barrier for transfer between these two basins of around 7 kcal/mol leads to 350 ns or longer stabilization of the bidentate structure in MD trajectories after careful equilibration of the protein. Although the barrier for substrate interconversion is substantially higher than k_B_T, it is still lower than the methyl transfer RDS, which is known to experimentally to have a free energy barrier around 18–19 kcal/mol. Although both binding modes have been remarked on, we have presented the first systematic determination of the free energy of these two binding modes.

Our work suggests the importance of charge transfer in methyltransferase modeling: as charge transfer is permitted between Mg^2+^ and the catecholate substrate in the increasingly large SQM regions, i) the free energy barrier is reduced by around 10 kcal/mol to bring it into consistency with experiment and ii) the non-bonded C-O distance of the Michaelis complex (2.7 Å) simultaneously comes into agreement with short distances (2.5–2.8 Å) observed experimentally in COMT crystal structures for the bidentate configuration. Future work will be aimed at using accelerated, fully first-principles methods to quantify differences in stability and reactivity of diverse substrate binding poses and protonation states in MTase active sites.

## Supporting Information

S1 Fig Bond distance distribution using different Mg^2+^ parameters. (TIF)

S2 Fig Bond distances from protocol 1. (TIF)

S3 Fig Bond distances from protocol 2. (TIF)

S4 Fig Bond distances from protocol 3. (TIF)

S5 Fig Bond distances from protocol 4. (TIF)

S6 Fig Dihedral angle distribution in restrained simulations. (TIF)

S7 Fig Oxygen charges and coordination environments for magnesium in bidentate and monodentate catecholcate (CAT) and dinitrocatecholate (DNC). Charges for CAT/DNC are obtained from RESP (HF/6-31G*) calculations as described in the text while the other charges are from the TIP3P or Amber ff12SB force fields.
(TIF)

S8 Fig Free energy of rearrangement (in kcal/mol) of catecholate from bidentate to alternate monodentate configuration. Error bars shown are from Monte Carlo analysis.
(TIF)

S9 Fig Representative, average bidentate (left) and monodentate (right) dinitrocatecholate (DNC) substrate configurations at the COMT active site. Substrates are shown in orange and labeled in brown while protein residues are shown in blue and labeled in dark blue. Key distances are labeled (in Å), except for D141-Mg^2+^, D1619-Mg^2+^, and Mg^2+^-H_2_O, which are omitted for clarity.
(TIF)

S10 Fig C-O distance distribution of alternate monodentate anionic oxygen and hydroxyl oxygen compared to standard monodentate and bidentate configurations. (TIF)

S11 Fig SQM region dependence of monodentate methyl transfer barriers. (TIF)

S12 Fig AM1/d and PM6 SMg free energy barrier comparison. (TIF)

S13 Fig Unconstrained SAM-CAT methyltransfer reaction coordinate computed with B3LYP and PM6. The pathway is obtained at the B3LYP/6-311++G* level of theory with nudged elastic band compared to single point energies obtained at the PM6 level of theory all treated with the COSMO implicit solvent model (ε = 10).
(TIF)

S14 Fig Constrained methyl transfer reaction coordinate for PM6 vs B3LYP. The (d(S-C)-d(C-O) in Å) reaction coordinate is obtained for PM6 and B3LYP/6–311++G* in COSMO implicit solvent (**ε** = 10).
(TIF)

S1 Table Mg^2+^ ion force field parameters. (DOCX)

S2 Table Dihedral angle and bond distances in restraints simulations with different force constant. (DOCX)

S3 Table Target d(S-C)-d(C-O) values for each window in umbrella sampling along with maximum and minimum values sampled for the bidentate configuration and SMgL semi-empirical model. (DOCX)

S4 Table Catecholate and dinitrocatecholate active site distances. (DOCX)

S5 Table SQM region dependence of monodentate and bidentate methyl transfer. (DOCX)

S6 Table SQM region dependence of geometric properties for the enzyme-substrate complex (ES) and transition state (TS). (DOCX)

S7 Table Comparison of reaction energies for methyltransfer to catecholate (CAT) from S-Adenosyl methionine (SAM) or trimethyl sulfonium (TMS) in the gas phase and with the COSMO solvation model (ε = 10 or ε = 78.4) computed with PM6 or B3LYP/6-311++G*. (DOCX)

S1 Text Extended discussion of equilibration protocols and force field parameters. (PDF)

S2 Text Discussion of PM6 vs. B3LYP reaction energies. (PDF)

S3 Text Description of MMPBSA. (PDF)

## References

[1] Senn HM, Thiel W. QM/MM methods for biomolecular systems. Angewandte Chemie International Edition. 2009;48(7):1198–229. 19173328 10.1002/anie.200802019

[2] Carvalho ATP, Barrozo A, Doron D, Kilshtain AV, Major DT, Kamerlin SCL. Challenges in computational studies of enzyme structure, function and dynamics. J Mol Graphics Modell. 2014;54:62–79. 10.1016/j.jmgm.2014.09.003 25306098

[3] Gao J, Truhlar DG. Quantum mechanical methods for enzyme kinetics. Annual Review of Physical Chemistry. 2002;53(1):467–505. 10.1146/annurev.physchem.53.091301.150114 11972016

[4] Martí S, Roca M, Andrés J, Moliner V, Silla E, Tuñón I, et al Theoretical insights in enzyme catalysis. Chemical Society Reviews. 2004;33(2):98–107. 14767505 10.1039/b301875j

[5] Warshel A, Levitt M. Theoretical studies of enzymic reactions: dielectric, electrostatic and steric stabilization of the carbonium ion in the reaction of lysozyme. J Mol Biol. 1976;103(2):227–49. 985660 10.1016/0022-2836(76)90311-9

[6] Hu H, Yang W. Free energies of chemical reactions in solution and in enzymes with ab initio QM/MM methods. Annual review of physical chemistry. 2008;59:573 10.1146/annurev.physchem.59.032607.093618 18393679 PMC3727228

[7] Xu D, Cui Q, Guo H. Quantum mechanical/molecular mechanical studies of zinc hydrolases. International Reviews in Physical Chemistry. 2014;33(1):1–41.

[8] van der Kamp MW, Mulholland AJ. Combined quantum mechanics/molecular mechanics (QM/MM) methods in computational enzymology. Biochemistry. 2013;52(16):2708–28. 10.1021/bi400215w 23557014

[9] Axelrod J, Tomchick R. Enzymatic O-methylation of epinephrine and other catechols. J Biol Chem. 1958;233(3):702–5. 13575440

[10] Cheng X, Kumar S, Posfai J, Pflugrath JW, Roberts RJ. Crystal structure of the Hhal DNA methyltransferase complexed with S-adenosyl-l-methionine. Cell. 1993;74(2):299–307. 8343957 10.1016/0092-8674(93)90421-l

[11] Labahn J, Granzin J, Schluckebier G, Robinson DP, Jack WE, Schildkraut I, et al Three-dimensional structure of the adenine-specific DNA methyltransferase M.Taq I in complex with the cofactor S-adenosylmethionine. Proc Natl Acad Sci U S A. 1994;91(23):10957–61. 7971991 10.1073/pnas.91.23.10957 PMC45145

[12] Lotta T, Vidgren J, Tilgmann C, Ulmanen I, Melen K, Julkunen I, et al Kinetics of Human Soluble and Membrane-Bound Catechol O-Methyltransferase: A Revised Mechanism and Description of the Thermolabile Variant of the Enzyme. Biochemistry. 1995;34(13):4202–10. 7703232 10.1021/bi00013a008

[13] Du X, Schwander M, Moresco EMY, Viviani P, Haller C, Hildebrand MS, et al A catechol-O-methyltransferase that is essential for auditory function in mice and humans. Proc Natl Acad Sci U S A. 2008;105(38):14609–14. 10.1073/pnas.0807219105 18794526 PMC2567147

[14] Coward JK, Slisz EP, Wu FYH. Kinetic studies on catechol O-methyltransferase, Product inhibition and the nature of the catechol binding site. Biochemistry. 1973;12(12):2291–7. 4736330 10.1021/bi00736a017

[15] Vidgren J, Svensson LA, Liljas A. Crystal structure of catechol O-methyltransferase. Nature. 1994;368(6469):354–8. 8127373 10.1038/368354a0

[16] Hegazi MF, Borchardt RT, Schowen RL.alpha.-Deuterium and carbon-13 isotope effects for methyl transfer catalyzed by catechol O-methyltransferase. SN2-like transition state. J Am Chem Soc. 1979;101(15):4359–65. 10.1021/ja00426a079 1262638

[17] Woodard RW, Tsai MD, Floss HG, Crooks PA, Coward JK. Stereochemical course of the transmethylation catalyzed by catechol O-methyltransferase. J Biol Chem. 1980;255(19):9124–7. 6997310

[18] Axelrod JJ. Methylation Reactions In The Formation And Metabolism Of Catecholamines And Other Biogenic Amines. Pharmacol Rev. 1966;18(1):95–113. 5323771

[19] Creveling CR, Dalgard N, Shimizu H, Daly JW. Catechol O-Methyltransferase: III. m- and p-O-Methylation of Catecholamines and Their Metabolites. Mol Pharmacol. 1970;6(6):691–6. 5497718

[20] Schultz E, Nissinen E. Inhibition of rat liver and duodenum soluble catechol-O-methyltransferase by a tight-binding inhibitor OR-462. Biochem Pharmacol. 1989;38(22):3953–6. 2597177 10.1016/0006-2952(89)90673-4

[21] Lautala P, Ulmanen I, Taskinen J. Molecular Mechanisms Controlling the Rate and Specificity of Catechol O-Methylation by Human Soluble Catechol O-Methyltransferase. Mol Pharmacol. 2001;59(2):393–402. 11160877 10.1124/mol.59.2.393

[22] Zhang J, Klinman JP. Enzymatic Methyl Transfer: Role of an Active Site Residue in Generating Active Site Compaction That Correlates with Catalytic Efficiency. J Am Chem Soc. 2011;133(43):17134–7. 10.1021/ja207467d 21958159 PMC3219439

[23] Mihel I, Knipe JO, Coward JK, Schowen RL.alpha.-Deuterium isotope effects and transition-state structure in an intramolecular model system for methyl-transfer enzymes. J Am Chem Soc. 1979;101(15):4349–51.

[24] Zhang J, Kulik HJ, Martinez TJ, Klinman JP. Mediation of donor–acceptor distance in an enzymatic methyl transfer reaction. Proc Natl Acad Sci U S A. 2015;112(26):7954–9. 10.1073/pnas.1506792112 26080432 PMC4491759

[25] Lau EY, Bruice TC. Importance of correlated motions in forming highly reactive near attack conformations in catechol O-methyltransferase. J Am Chem Soc. 1998;120(48):12387–94.

[26] Lau EY, Bruice TC. Comparison of the Dynamics for Ground-State and Transition-State Structures in the Active Site of Catechol O-Methyltransferase. J Am Chem Soc. 2000;122(30):7165–71.

[27] Kuhn B, Kollman PA. QM−FE and Molecular Dynamics Calculations on Catechol O-Methyltransferase: Free Energy of Activation in the Enzyme and in Aqueous Solution and Regioselectivity of the Enzyme-Catalyzed Reaction. J Am Chem Soc. 2000;122(11):2586–96.

[28] Zheng YJ, Bruice TC. A theoretical examination of the factors controlling the catalytic efficiency of a transmethylation enzyme: Catechol O-methyltransferase. J Am Chem Soc. 1997;119(35):8137–45.

[29] Rutherford K, Bennion BJ, Parson WW, Daggett V. The 108M Polymorph of Human Catechol O-Methyltransferase Is Prone to Deformation at Physiological Temperatures†. Biochemistry. 2006;45(7):2178–88. 16475806 10.1021/bi051988i

[30] Rutherford K, Daggett V. A Hotspot of Inactivation: The A22S and V108M Polymorphisms Individually Destabilize the Active Site Structure of Catechol O-Methyltransferase. Biochemistry. 2009;48(27):6450–60. 10.1021/bi900174v 19435324 PMC2906713

[31] Tsao D, Diatchenko L, Dokholyan NV. Structural Mechanism of S-Adenosyl Methionine Binding to Catechol O-Methyltransferase. PLoS ONE. 2011;6(8):e24287 10.1371/journal.pone.0024287 21904625 PMC3164188

[32] Lameira J, Bora RP, Chu ZT, Warshel A. Methyltransferases do not work by compression, cratic, or desolvation effects, but by electrostatic preorganization. Proteins: Struct, Funct, Bioinf. 2015;83(2):318–30. 10.1002/prot.24717 PMC4300294 25388538

[33] Roca M, Martí S, Andrés J, Moliner V, Tuñón I, Bertrán J, et al Theoretical modeling of enzyme catalytic power: analysis of “cratic” and electrostatic factors in catechol O-methyltransferase. J Am Chem Soc. 2003;125(25):7726–37. 12812514 10.1021/ja0299497

[34] Kanaan N, Ruiz Pernia JJ, Williams IH. QM/MM simulations for methyl transfer in solution and catalysed by COMT: ensemble-averaging of kinetic isotope effects. Chem Commun (Cambridge, U K). 2008;(46):6114–6. 10.1039/b814212b 19082090

[35] Kulik HJ, Drennan CL. Substrate Placement Influences Reactivity in Non-heme Fe (II) Halogenases and Hydroxylases. J Biol Chem. 2013;288:11233–41. 10.1074/jbc.M112.415570 23449977 PMC3630895

[36] Martinie RJ, Livada J, Chang W-c, Green MT, Krebs C, Bollinger JM, et al Experimental Correlation of Substrate Position with Reaction Outcome in the Aliphatic Halogenase, SyrB2. J Am Chem Soc. 2015. 10.1021/jacs.5b03370 PMC4456221 25965587

[37] Rod TH, Ryde U. Quantum Mechanical Free Energy Barrier for an Enzymatic Reaction. Phys Rev Lett. 2005;94(13):138302 15904045 10.1103/PhysRevLett.94.138302

[38] Rod TH, Ryde U. Accurate QM/MM Free Energy Calculations of Enzyme Reactions: Methylation by Catechol O-Methyltransferase. J Chem Theory Comput. 2005;1(6):1240–51. 10.1021/ct0501102 26631668

[39] Roca M, Moliner V, Tuñón I, Hynes JT. Coupling between Protein and Reaction Dynamics in Enzymatic Processes: Application of Grote−Hynes Theory to Catechol O-Methyltransferase. J Am Chem Soc. 2006;128(18):6186–93. 16669689 10.1021/ja058826u

[40] Ruggiero GD, Williams IH, Roca M, Moliner V, Tuñón I. QM/MM Determination of Kinetic Isotope Effects for COMT-Catalyzed Methyl Transfer Does Not Support Compression Hypothesis. J Am Chem Soc. 2004;126(28):8634–5. 15250699 10.1021/ja048055e

[41] Case DA, Berryman JT, Betz RM, Cerutti DS, Cheatham TE III, Darden TA, et al AMBER 2015, University of California, San Francisco 2015.

[42] Salomon-Ferrer R, Case DA, Walker RC. An overview of the Amber biomolecular simulation package. Wiley Interdiscip Rev: Comput Mol Sci. 2013;3(2):198–210.

[43] Rutherford K, Le Trong I, Stenkamp RE, Parson WW. Crystal Structures of Human 108V and 108M Catechol O-Methyltransferase. J Mol Biol. 2008;380(1):120–30. 10.1016/j.jmb.2008.04.040 18486144

[44] Maier JA, Martinez C, Kasavajhala K, Wickstrom L, Hauser KE, Simmerling C. ff14SB: Improving the Accuracy of Protein Side Chain and Backbone Parameters from ff99SB. J Chem Theory Comput. 2015;11(8):3696–713. 10.1021/acs.jctc.5b00255 26574453 PMC4821407

[45] Hornak V, Abel R, Okur A, Strockbine B, Roitberg A, Simmerling C. Comparison of multiple Amber force fields and development of improved protein backbone parameters. Proteins: Struct, Funct, Bioinf. 2006;65(3):712–25. 10.1002/prot.21123 PMC4805110 16981200

[46] Wang J, Wolf RM, Caldwell JW, Kollman PA, Case DA. Development and testing of a general amber force field. J Comput Chem. 2004;25(9):1157–74. 15116359 10.1002/jcc.20035

[47] Bayly CI, Cieplak P, Cornell W, Kollman PA. A well-behaved electrostatic potential based method using charge restraints for deriving atomic charges: the RESP model. J Phys Chem. 1993;97(40):10269–80.

[48] Gordon MS, Schmidt MW. Advances in electronic structure theory: GAMESS a decade later. Theory Appl Comput Chem: First Forty Years. 2005:1167–89.

[49] Harihara PC, Pople JA. Influence of Polarization Functions on Molecular-Orbital Hydrogenation Energies. Theor Chim Acta. 1973;28(3):213–22.

[50] Wang F, Becker J-P, Cieplak P, Dupradeau F-Y. R.E.D. Python: Object oriented programming for Amber force fields, Université de Picardie—Jules Verne, Sanford|Burnham Medical Research Institute, Nov. 2013 [6/16/15]. Available from: http://q4md-forcefieldtools.org/REDServer-Development/.

[51] Vanquelef E, Simon S, Marquant G, Garcia E, Klimerak G, Delepine JC, et al R.E.D. Server: a web service for deriving RESP and ESP charges and building force field libraries for new molecules and molecular fragments. Nucleic Acids Res. 2011;39(suppl 2):W511–W7. 21609950 10.1093/nar/gkr288 PMC3125739

[52] Dupradeau F-Y, Pigache A, Zaffran T, Savineau C, Lelong R, Grivel N, et al The R.E.D. tools: advances in RESP and ESP charge derivation and force field library building. Phys Chem Chem Phys. 2010;12(28):7821–39. 10.1039/c0cp00111b 20574571 PMC2918240

[53] Allnér O, Nilsson L, Villa A. Magnesium Ion–Water Coordination and Exchange in Biomolecular Simulations. J Chem Theory Comput. 2012;8(4):1493–502. 10.1021/ct3000734 26596759

[54] H++ server v3.1. [6/16/2015]. Available from: http://biophysics.cs.vt.edu/H++.

[55] Anandakrishnan R, Aguilar B, Onufriev AV. H++ 3.0: automating pK prediction and the preparation of biomolecular structures for atomistic molecular modeling and simulations. Nucleic Acids Res. 2012;40(W1):W537–W41. 22570416 10.1093/nar/gks375 PMC3394296

[56] Gordon JC, Myers JB, Folta T, Shoja V, Heath LS, Onufriev A. H++: a server for estimating pKas and adding missing hydrogens to macromolecules. Nucleic Acids Res. 2005;33(suppl 2):W368–W71. 15980491 10.1093/nar/gki464 PMC1160225

[57] Myers J, Grothaus G, Narayanan S, Onufriev A. A simple clustering algorithm can be accurate enough for use in calculations of pKs in macromolecules. Proteins: Struct, Funct, Bioinf. 2006;63(4):928–38. 10.1002/prot.20922 16493626

[58] Jorgensen WL, Chandrasekhar J, Madura JD, Impey RW, Klein ML. Comparison of simple potential functions for simulating liquid water. J Chem Phys. 1983;79(2):926–35.

[59] Ryckaert J-P, Ciccotti G, Berendsen HJC. Numerical integration of the cartesian equations of motion of a system with constraints: molecular dynamics of n-alkanes. J Comput Phys. 1977;23(3):327–41.

[60] Souaille M, Roux Bt. Extension to the weighted histogram analysis method: combining umbrella sampling with free energy calculations. Comput Phys Commun. 2001;135(1):40–57.

[61] Kumar S, Rosenberg JM, Bouzida D, Swendsen RH, Kollman PA. THE weighted histogram analysis method for free-energy calculations on biomolecules. I. The method. J Comput Chem. 1992;13(8):1011–21.

[62] Grossfield A. "WHAM: the weighted histogram analysis method", version 2.0.9 [06/15/2015]. Available from: http://membrane.urmc.rochester.edu/content/wham.

[63] Torrie GM, Valleau JP. Nonphysical sampling distributions in Monte Carlo free-energy estimation: Umbrella sampling. J Comput Phys. 1977;23(2):187–99.

[64] Dewar MJS, Zoebisch EG, Healy EF, Stewart JJP. Development and use of quantum mechanical molecular models. 76. AM1: a new general purpose quantum mechanical molecular model. J Am Chem Soc. 1985;107(13):3902–9.

[65] Stewart JJP. Optimization of parameters for semiempirical methods V: Modification of NDDO approximations and application to 70 elements. J Mol Model. 2007;13(12):1173–213. 17828561 10.1007/s00894-007-0233-4 PMC2039871

[66] Lee C, Yang W, Parr RG. Development of the Colle-Salvetti correlation-energy formula into a functional of the electron density. Phys Rev B. 1988;37(2):785–9. 10.1103/physrevb.37.785 9944570

[67] Becke AD. Density‐functional thermochemistry. III. The role of exact exchange. J Chem Phys. 1993;98(7):5648–52.

[68] Stephens PJ, Devlin FJ, Chabalowski CF, Frisch MJ. Ab Initio Calculation of Vibrational Absorption and Circular Dichroism Spectra Using Density Functional Force Fields. J Phys Chem. 1994;98(45):11623–7.

[69] Miller BR III, McGee TD Jr, Swails JM, Homeyer N, Gohlke H, Roitberg AE. MMPBSA. py: an efficient program for end-state free energy calculations. J Chem Theory Comput. 2012;8(9):3314–21. 10.1021/ct300418h 26605738

[70] Massova I, Kollman PA. Combined molecular mechanical and continuum solvent approach (MM-PBSA/GBSA) to predict ligand binding. Perspect Drug Discovery Des. 2000;18(1):113–35.

[71] Sharp KA, Honig B. Calculating total electrostatic energies with the nonlinear Poisson-Boltzmann equation. J Phys Chem. 1990;94(19):7684–92.

[72] Tsui V, Case DA. Theory and applications of the generalized born solvation model in macromolecular simulations. Biopolymers. 2000;56(4):275–91. 11754341 10.1002/1097-0282(2000)56:4<275::AID-BIP10024>3.0.CO;2-E

[73] Onufriev A, Bashford D, Case DA. Exploring protein native states and large‐scale conformational changes with a modified generalized born model. Proteins: Struct, Funct, Bioinf. 2004;55(2):383–94. 10.1002/prot.20033 15048829

[74] Hou T, Wang J, Li Y, Wang W. Assessing the Performance of the MM/PBSA and MM/GBSA Methods. 1. The Accuracy of Binding Free Energy Calculations Based on Molecular Dynamics Simulations. J Chem Inf Model. 2011;51(1):69–82. 10.1021/ci100275a 21117705 PMC3029230

[75] Bonifácio MJ, Archer M, Rodrigues ML, Matias PM, Learmonth DA, Carrondo MA, et al Kinetics and Crystal Structure of Catechol-O-Methyltransferase Complex with Co-Substrate and a Novel Inhibitor with Potential Therapeutic Application. Mol Pharmacol. 2002;62(4):795–805. 12237326 10.1124/mol.62.4.795

[76] Palma PN, Rodrigues ML, Archer M, Bonifácio MJ, Loureiro AI, Learmonth DA, et al Comparative Study of ortho- and meta-Nitrated Inhibitors of Catechol-O-methyltransferase: Interactions with the Active Site and Regioselectivity of O-Methylation. Mol Pharmacol. 2006;70(1):143–53. 16618795 10.1124/mol.106.023119

[77] Tsuji E, Okazaki K, Takeda K. Crystal structures of rat catechol-O-methyltransferase complexed with coumarine-based inhibitor. Biochem Biophys Res Commun. 2009;378(3):494–7. 10.1016/j.bbrc.2008.11.085 19056347

[78] Ellermann M, Lerner C, Burgy G, Ehler A, Bissantz C, Jakob-Roetne R, et al Catechol-O-methyltransferase in complex with substituted 3'-deoxyribose bisubstrate inhibitors. Acta Crystallogr Sect D-Biol Crystallogr. 2012;68(3):253–60. 22349227 10.1107/S0907444912001138

[79] Harrison ST, Poslusney MS, Mulhearn JJ, Zhao Z, Kett NR, Schubert JW, et al Synthesis and Evaluation of Heterocyclic Catechol Mimics as Inhibitors of Catechol-O-methyltransferase (COMT). ACS Med Chem Lett. 2015;6(3):318–23. 10.1021/ml500502d 25815153 PMC4360154

[80] Tsao D, Liu S, Dokholyan NV. Regioselectivity of catechol O-methyltransferase confers enhancement of catalytic activity. Chem Phys Lett. 2011;506(4–6):135–8. 21731105 10.1016/j.cplett.2011.03.048 PMC3125089

[81] Gerhards M, Perl W, Schumm S, Henrichs U, Jacoby C, Kleinermanns K. Structure and vibrations of catechol and catechol⋅H2O(D2O) in the S0 and S1 state. J Chem Phys. 1996;104(23):9362–75.

[82] Dudev T, Lim C. Principles Governing Mg, Ca, and Zn Binding and Selectivity in Proteins. Chem Rev. 2003;103(3):773–88. 12630852 10.1021/cr020467n

[83] Bleuzen A, Pittet PA, Helm L, Merbach AE. Water exchange on magnesium (II) in aqueous solution: a variable temperature and pressure 17O NMR study. Mag Reson Chem. 1997;35(11):765–73.

[84] Roca M, Andrés J, Moliner V, Tuñón I, Bertrán J. On the Nature of the Transition State in Catechol O-Methyltransferase. A Complementary Study Based on Molecular Dynamics and Potential Energy Surface Explorations. J Am Chem Soc. 2005;127(30):10648–55. 16045352 10.1021/ja051503d

[85] Sparta M, Alexandrova AN. How Metal Substitution Affects the Enzymatic Activity of Catechol-O-Methyltransferase. PLoS ONE. 2012;7(10):e47172 10.1371/journal.pone.0047172 23056605 PMC3466255

[86] Furuki T, Sakurai M, Inoue Y. An application of the reaction field theory to hydrated metal cations in the framework of the MNDO, AM1, and PM3 methods. J Comput Chem. 1995;16(3):378–84.

[87] Garcia-Viloca M, Gao J, Karplus M, Truhlar DG. How enzymes work: analysis by modern rate theory and computer simulations. Science. 2004;303(5655):186–95. 14716003 10.1126/science.1088172

[88] Cao Y, Chen Z-J, Jiang H-D, Chen J-Z. Computational Studies of the Regioselectivities of COMT-Catalyzed Meta-/Para-O Methylations of Luteolin and Quercetin. J Phys Chem B. 2014;118(2):470–81. 10.1021/jp410296s 24354565

[89] Bai H-W, Shim J-Y, Yu J, Zhu BT. Biochemical and Molecular Modeling Studies of the O-Methylation of Various Endogenous and Exogenous Catechol Substrates Catalyzed by Recombinant Human Soluble and Membrane-Bound Catechol-O-Methyltransferases†. Chem Res Toxicol. 2007;20(10):1409–25. 17880176 10.1021/tx700174w

[90] Tsuji E, Okazaki K, Isaji M, Takeda K. Crystal structures of the Apo and Holo form of rat catechol-O-methyltransferase. J Struct Biol. 2009;165(3):133–9. 10.1016/j.jsb.2008.11.012 19111934

[91] Ehler A, Benz J, Schlatter D, Rudolph MG. Mapping the conformational space accessible to catechol-O-methyltransferase. Acta Crystallogr Sect D-Biol Crystallogr. 2014;70(8):2163–74. 25084335 10.1107/S1399004714012917 PMC4118827

[92] Rutherford K, Alphandéry E, McMillan A, Daggett V, Parson WW. The V108M mutation decreases the structural stability of catechol O-methyltransferase. Biochim Biophys Acta, Proteins Proteomics. 2008;1784(7–8):1098–105. 10.1016/j.bbapap.2008.04.006 18474266

