# Supplementary figures and images for: Computational Investigation of the Interplay of Substrate Positioning and Reactivity in Catechol O-Methyltransferase
Source: PLoS One. 2016 Aug 26;11(8):e0161868. doi: 10.1371/journal.pone.0161868 (PMC5001633; doi:10.1371/journal.pone.0161868)

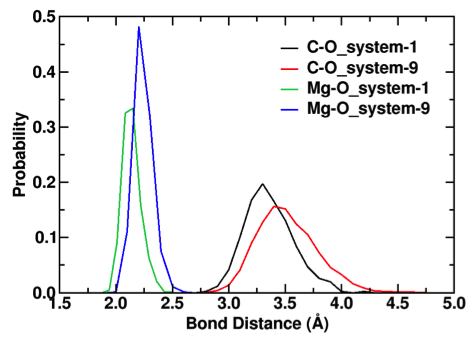

Supplement: S1 Fig — (TIF) [file pone.0161868.s001.tif]

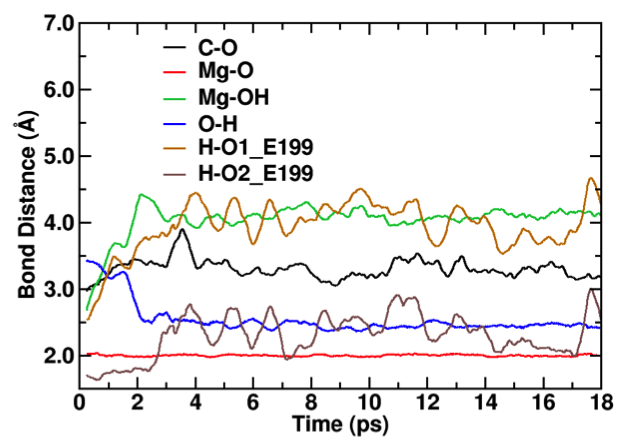

Supplement: S2 Fig — (TIF) [file pone.0161868.s002.tif]

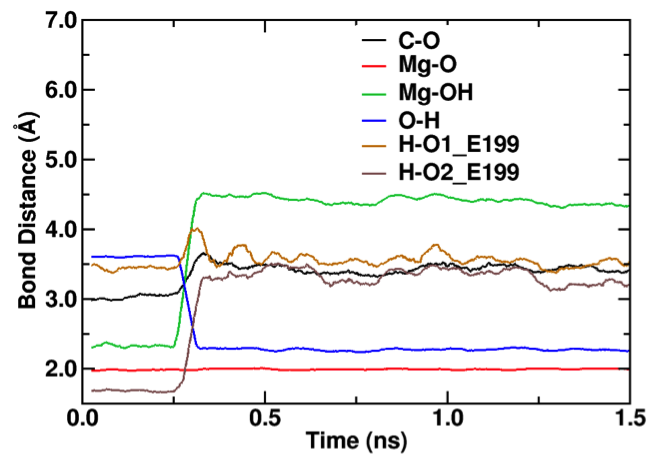

Supplement: S3 Fig — (TIF) [file pone.0161868.s003.tif]

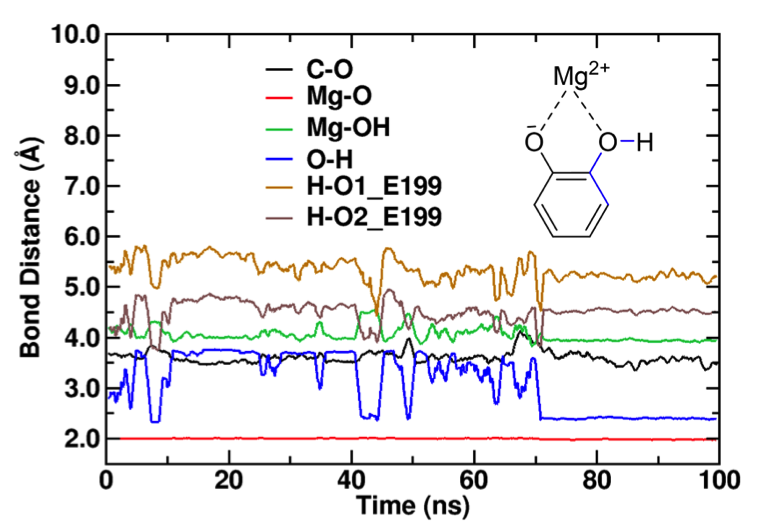

Supplement: S4 Fig — (TIF) [file pone.0161868.s004.tif]

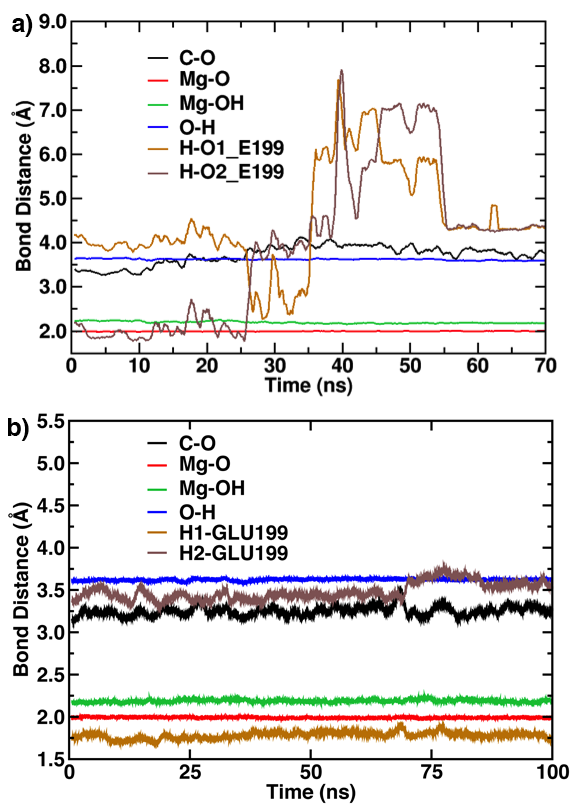

Supplement: S5 Fig — (TIF) [file pone.0161868.s005.tif]

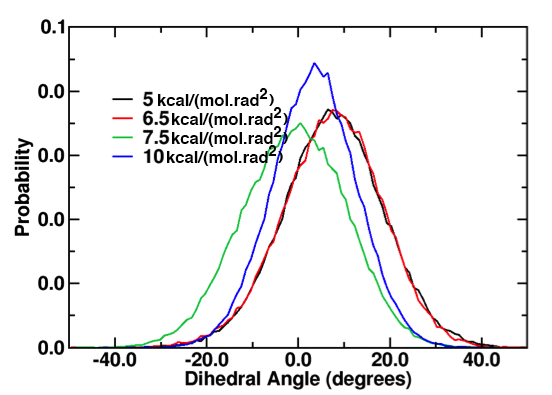

Supplement: S6 Fig — (TIF) [file pone.0161868.s006.tif]

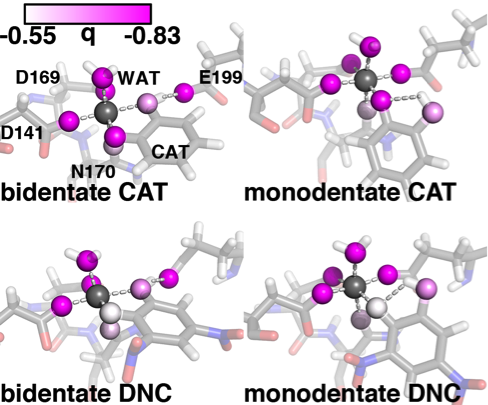

Supplement: S7 Fig — Charges for CAT/DNC are obtained from RESP (HF/6-31G*) calculations as described in the text while the other charges are from the TIP3P or Amber ff12SB force fields. (TIF) [file pone.0161868.s007.tif]

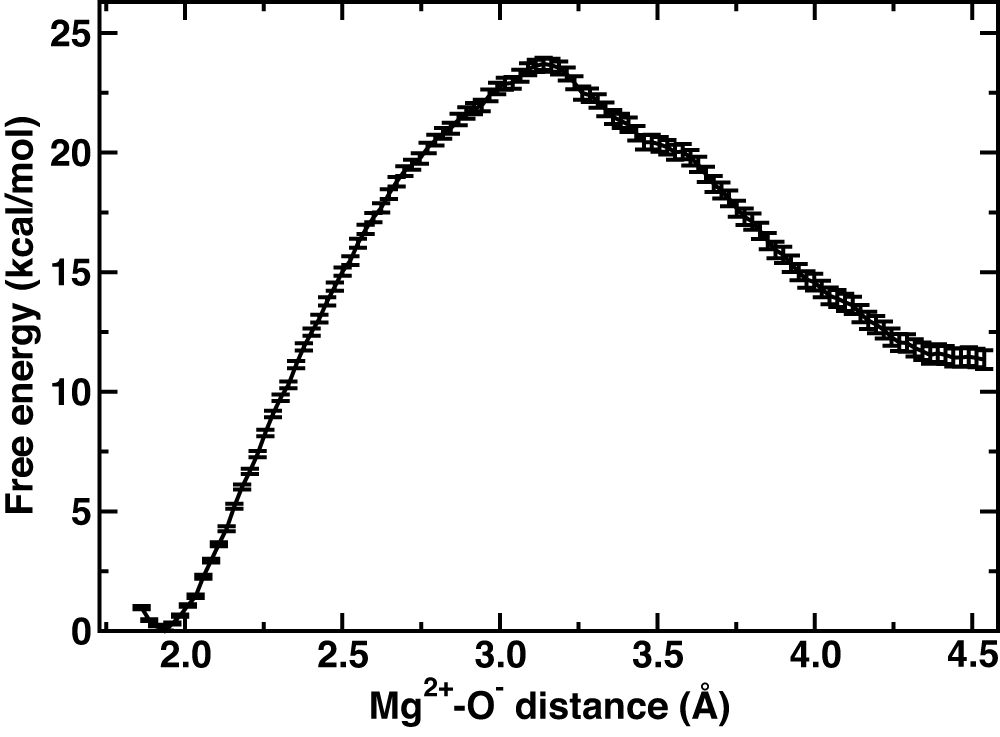

Supplement: S8 Fig — Error bars shown are from Monte Carlo analysis. (TIF) [file pone.0161868.s008.tif]

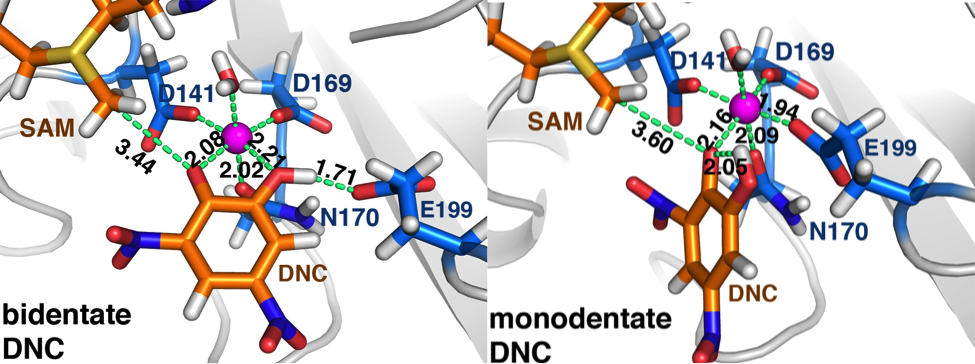

Supplement: S9 Fig — Substrates are shown in orange and labeled in brown while protein residues are shown in blue and labeled in dark blue. Key distances are labeled (in Å), except for D141-Mg2+, D1619-Mg2+, and Mg2+-H2O, which are omitted for clarity. (TIF) [file pone.0161868.s009.tif]

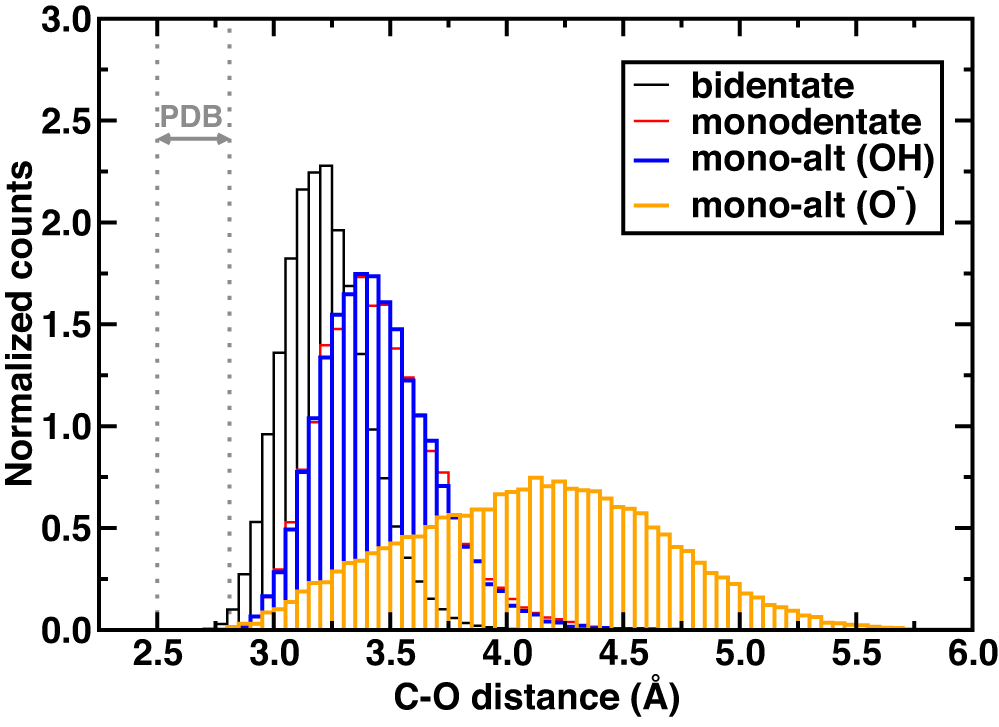

Supplement: S10 Fig — (TIF) [file pone.0161868.s010.tif]

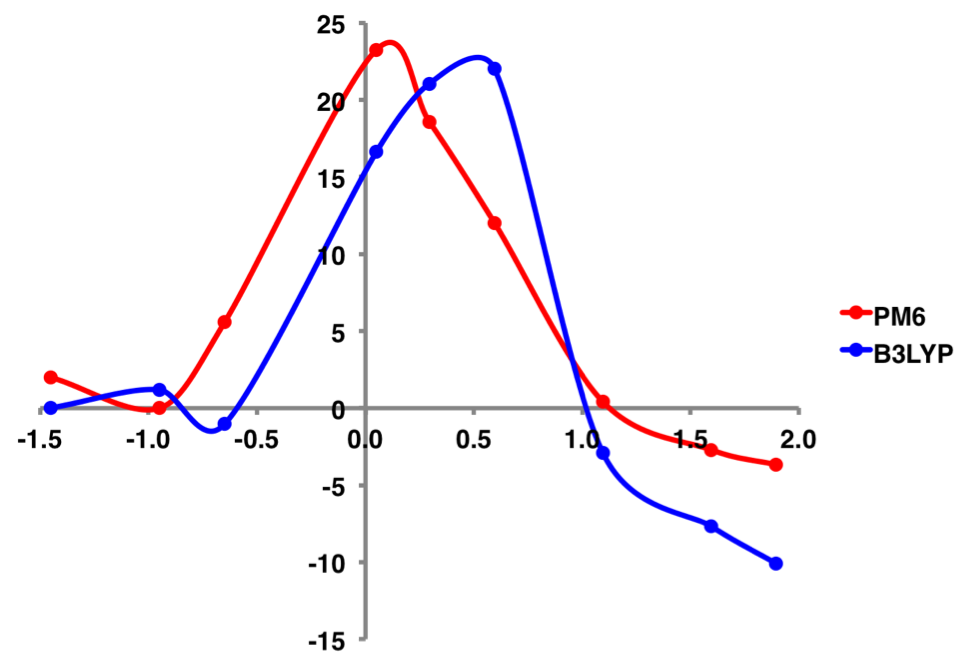

Supplement: S11 Fig — (TIF) [file pone.0161868.s011.tif]

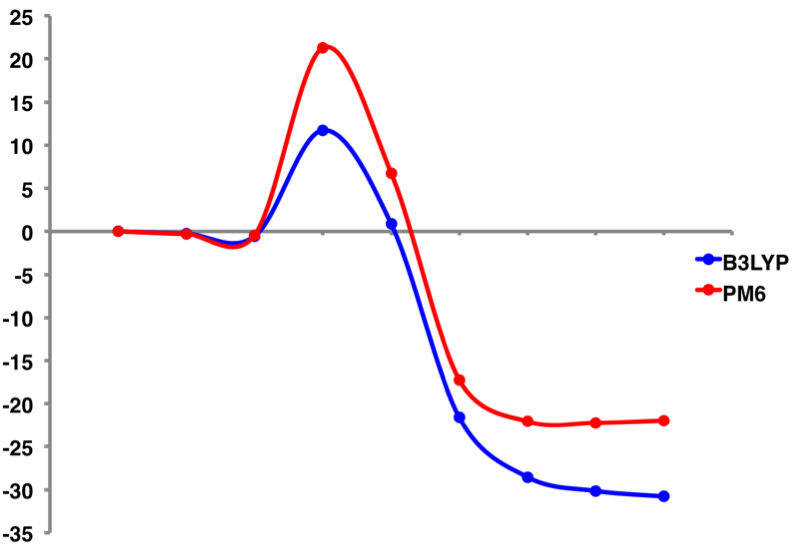

Supplement: S12 Fig — (TIF) [file pone.0161868.s012.tif]

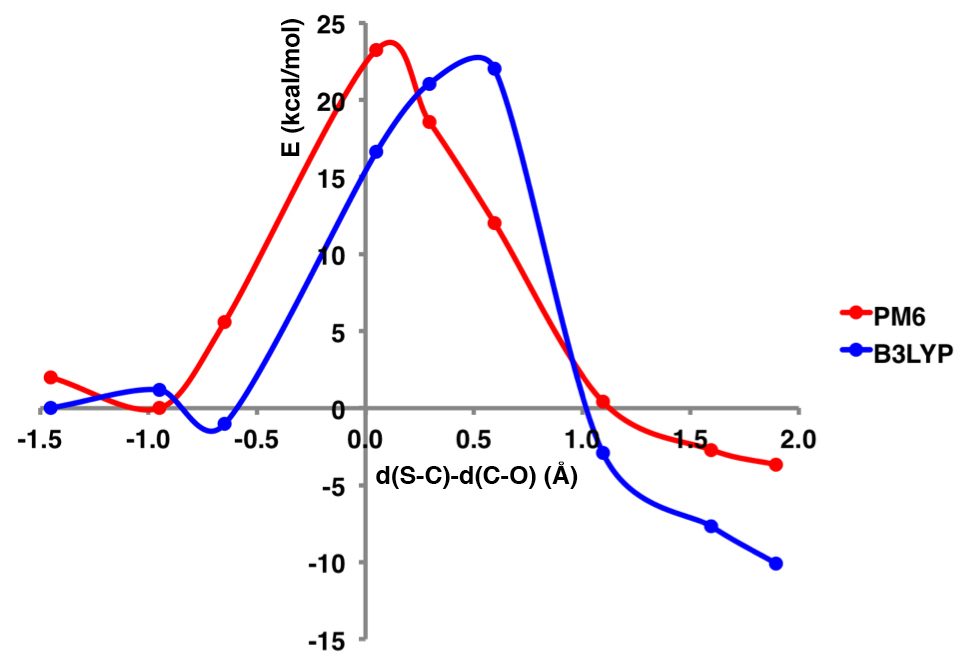

Supplement: S13 Fig — The pathway is obtained at the B3LYP/6-311++G* level of theory with nudged elastic band compared to single point energies obtained at the PM6 level of theory all treated with the COSMO implicit solvent model (ε = 10). (TIF) [file pone.0161868.s013.tif]

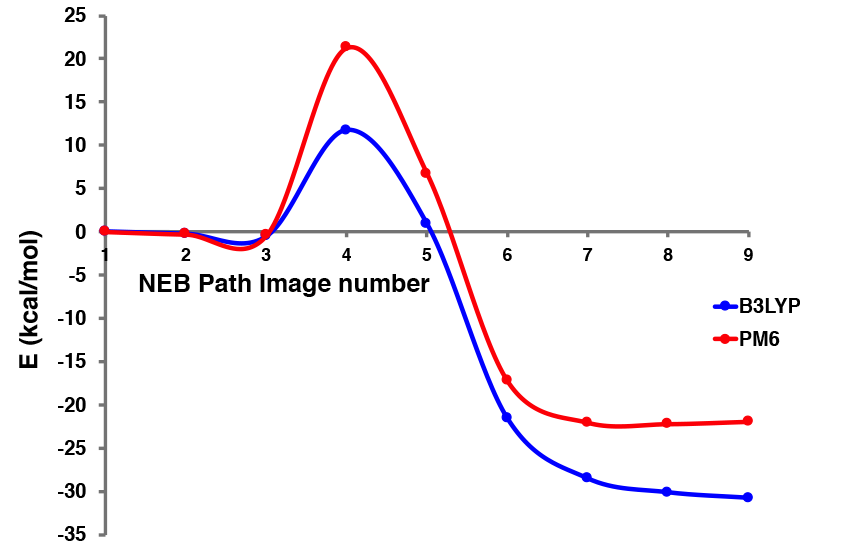

Supplement: S14 Fig — The (d(S-C)-d(C-O) in Å) reaction coordinate is obtained for PM6 and B3LYP/6–311++G* in COSMO implicit solvent (ε = 10). (TIF) [file pone.0161868.s014.tif]
