# Supplementary material for: Computational Investigation of the Interplay of Substrate Positioning and Reactivity in Catechol O-Methyltransferase
Source: PLoS One. 2016 Aug 26;11(8):e0161868. doi: 10.1371/journal.pone.0161868 (PMC5001633; doi:10.1371/journal.pone.0161868)
Supplement: S1 Table — (DOCX) [file pone.0161868.s015.docx]

| vDW radius (Å)/  ε (kcal/mol) | 1.554 | 1.495 | 1.437 |
| --- | --- | --- | --- |
| 0.002950000 | 1^1^ | 2 | 3 |
| 0.012276481 | 4 | 5 | 6 |
| 0.02257962 | 7 | 8 | 9^2^ |

^1^Allnér, O.; Nilsson, L.; and Villa, A., Magnesium Ion–Water Coordination and Exchange in Biomolecular Simulations. *J. Chem. Theory Comput.* **2012**, *8*, 1493-1502.

^2^Li, P. and Merz, K. M. Jr. Taking into Account the Ion-Induced Dipole Interaction in the Nonbonded Model of Ions .*J. Chem. Theory Comput.* 2014, 10, 289-297.
