# Supplementary material for: Computational Investigation of the Interplay of Substrate Positioning and Reactivity in Catechol O-Methyltransferase
Source: PLoS One. 2016 Aug 26;11(8):e0161868. doi: 10.1371/journal.pone.0161868 (PMC5001633; doi:10.1371/journal.pone.0161868)
Supplement: S2 Table — (DOCX) [file pone.0161868.s016.docx]

| Force constant (kcal.mol^-1^.rad^-2^) | Target Dihedral Angle (degree) | FWHM  of Dihedral  (degree) | C-O Distance  (Å) | O-Mg Distance  (Å) | OH-Mg Distance  (Å) | O-H Distance (Å) |
| --- | --- | --- | --- | --- | --- | --- |
|  |  |  | mean | mean | mean | mean |
| 5.0 | -5.5 | 24 | 3.34 | 2.06 | 3.27 | 3.66 |
| 6.5 | -5.5 | 24 | 3.26 | 2.05 | 3.57 | 3.70 |
| 7.5 | -5.5 | 27 | 3.58 | 2.06 | 3.54 | 3.69 |
| 10.0 | -5.5 | 21 | 3.26 | 2.07 | 3.38 | 3.68 |
