# Supplementary material for: Computational Investigation of the Interplay of Substrate Positioning and Reactivity in Catechol O-Methyltransferase
Source: PLoS One. 2016 Aug 26;11(8):e0161868. doi: 10.1371/journal.pone.0161868 (PMC5001633; doi:10.1371/journal.pone.0161868)
Supplement: S3 Table — (DOCX) [file pone.0161868.s017.docx]

| # | Target (Å) | k (kcal/(mol•Å^2^)) | Max.  (Å) | Min. (Å) | Width (Å) |
| --- | --- | --- | --- | --- | --- |
| 1 | -1.15 | 10 | -0.70 | -1.47 | 0.77 |
| 2 | -0.40 | 20 | -0.33 | -0.98 | 0.65 |
| 3 | 0.10 | 40 | 0.06 | -0.52 | 0.58 |
| 4 | 0.00 | 240 | 0.11 | -0.20 | 0.31 |
| 5 | 0.15 | 240 | 0.26 | -0.05 | 0.31 |
| 6 | 0.30 | 240 | 0.43 | 0.15 | 0.29 |
| 7 | 0.45 | 160 | 0.68 | 0.32 | 0.36 |
| 8 | 0.65 | 40 | 1.23 | 0.67 | 0.56 |
| 9 | 0.90 | 40 | 1.33 | 0.86 | 0.47 |
| 10 | 1.55 | 10 | 2.10 | 1.23 | 0.86 |
