# Supplementary material for: Computational Investigation of the Interplay of Substrate Positioning and Reactivity in Catechol O-Methyltransferase
Source: PLoS One. 2016 Aug 26;11(8):e0161868. doi: 10.1371/journal.pone.0161868 (PMC5001633; doi:10.1371/journal.pone.0161868)
Supplement: S4 Table — (DOCX) [file pone.0161868.s018.docx]

|  | Distances (Å) | | | |
| --- | --- | --- | --- | --- |
|  | CAT | | DNC | |
| interaction | bidentate | monodentate | bidentate | monodentate |
| Mg^2+^-D141 | 1.93 | 1.98 | 1.90 | 1.93 |
| Mg^2+^-D169 | 1.92 | 1.99 | 1.94 | 1.94 |
| Mg^2+^-N170 | 2.13 | 2.09 | 2.02 | 2.09 |
| Mg^2+^-H_2_O | 2.12 | 2.07 | 2.05 | 2.08 |
| Mg^2+^-sub(O^-^) | 1.97 | 1.94 | 2.08 | 2.16 |
| Mg^2+^-sub(OH) | 2.15 |  | 2.21 |  |
| sub(H)-E199 | 1.77 |  | 1.71 |  |
| Mg^2+^-E199 |  | 2.00 |  | 1.94 |
| SAM-sub(O^-^) | 3.23 | 3.40 | 3.44 | 3.60 |
