# Supplementary material for: Computational Investigation of the Interplay of Substrate Positioning and Reactivity in Catechol O-Methyltransferase
Source: PLoS One. 2016 Aug 26;11(8):e0161868. doi: 10.1371/journal.pone.0161868 (PMC5001633; doi:10.1371/journal.pone.0161868)
Supplement: S5 Table — (DOCX) [file pone.0161868.s019.docx]

|  | ΔG^‡^ (kcal/mol) | ΔG_Rxn_ (kcal/mol) |
| --- | --- | --- |
| mono-S | 30.8 | 13.5 |
| mono-SMg | 28.7 | 11.5 |
| bide-S | 32.1 | 15.6 |
| bide-SMg | 28.1 | 9.2 |
