# Supplementary material for: Computational Investigation of the Interplay of Substrate Positioning and Reactivity in Catechol O-Methyltransferase
Source: PLoS One. 2016 Aug 26;11(8):e0161868. doi: 10.1371/journal.pone.0161868 (PMC5001633; doi:10.1371/journal.pone.0161868)
Supplement: S6 Table — (DOCX) [file pone.0161868.s020.docx]

|  | S-C (Å) | C-O (Å) | Δ (Å) | ∠S-C-O (°) |
| --- | --- | --- | --- | --- |
| **ES** | | | | |
| mono-S | 1.78 | 3.38 | -1.60 | 157.3 |
| mono-SMg | 1.77 | 3.22 | -1.45 | 155.7 |
| bide-S | 1.76 | 3.16 | -1.40 | 153.0 |
| bide-SMg | 1.76 | 2.98 | -1.22 | 149.3 |
| **TS** | | | | |
| mono-S | 2.27 | 1.88 | 0.39 | 171.1 |
| mono-SMg | 2.25 | 1.83 | 0.42 | 169.8 |
| bide-S | 2.28 | 1.88 | 0.40 | 171.6 |
| bide-SMg | 2.24 | 1.85 | 0.39 | 170.9 |
