# Supplementary material for: Computational Investigation of the Interplay of Substrate Positioning and Reactivity in Catechol O-Methyltransferase
Source: PLoS One. 2016 Aug 26;11(8):e0161868. doi: 10.1371/journal.pone.0161868 (PMC5001633; doi:10.1371/journal.pone.0161868)
Supplement: S7 Table — (DOCX) [file pone.0161868.s021.docx]

|  | Gas phase | | | ε=10 | | | ε=78.4 | | |
| --- | --- | --- | --- | --- | --- | --- | --- | --- | --- |
|  | PM6 | B3LYP | Diff. | PM6 | B3LYP | Diff. | PM6 | B3LYP | Diff. |
| CAT+SAM | -59.2 | -77.6 | -18.4 | -13.7 | -30.6 | -16.9 | -3.6 | -24.7 | -21.1 |
| CAT+TMS | -110.8 | -132.0 | -21.2 | -11.9 | -31.0 | -19.1 | 2.3 | -20.7 | -23.1 |
