# Supplementary material for: Computational Investigation of the Interplay of Substrate Positioning and Reactivity in Catechol O-Methyltransferase
Source: PLoS One. 2016 Aug 26;11(8):e0161868. doi: 10.1371/journal.pone.0161868 (PMC5001633; doi:10.1371/journal.pone.0161868)
Supplement: S1 Text — (PDF) [file pone.0161868.s022.pdf]

I. Classical MD protocols

I.A. Mg^{2+} ion parameters selection

The results of any simulation are likely to be sensitive to the choice of Mg^{2+} parameters. We have simulated nine combinations of Mg^{2+} parameters spanning two previously published suggestions in the literature[1-2] (Table S1). The test system contains one Mg^{2+} ion, S-Adenosyl-L-methionine (SAM), and one dinitrocatecholate anion (DNC) in a 40 Å cubic box of TIP3P water. SAM and DNC parameters are described by the general Amber force field (GAFF) with partial charges assigned from restrained electrostatic potential charges obtained with GAMESS-US at the Hartree-Fock level using 6-31G* basis set as implemented by the R.E.D.S. webserver. Initially, the Mg^{2+} ion and dinitrocatecholate ion were placed in such a way that both DNC oxygen atoms were coordinated by the Mg^{2+} ion. One chloride ion was added to neutralize the system. All classical molecular dynamics simulations were carried out using GPU-accelerated version of PMEMD.

Each system was minimized for 5000 steepest descent steps followed by 10000 conjugate gradient steps. During minimization, positional restraints were applied on the dinitrocatecholate and Mg^{2+} ion (200 kcal/(mol-Å^{-2})). Next, the system was heated to T = 300 K in the NVT ensemble for 20 ps with a positional restraint still on the dinitrocatecholate and Mg^{2+} ion. Next, an NPT equilibration run was carried out for 2 ns without any restraints. Finally, a 20 ns NPT production run was carried out for each system. The corresponding nine trajectories were analyzed for system properties.

In order to evaluate the best force field parameters, we compared the SAM carbon – dinitrocatecholate oxygen (anion) distance (C-O) and Mg-O$^-$ bond distances for all cases. As shown in the Figure S6, the C-O and Mg-O$^-$ bond distances distributions are determined from the

simulated trajectories of system 1 and system 9. Both bond distances are shorter when using set 1 Mg^{2+} ion parameters compared to set 9 parameters. Results from parameter sets 2-8 were intermediate between these two cases and not shown. Using set 1 parameters ensures no exchange of water molecules over the course of simulation, commensurate with experimental exchange rates for water molecules coordinating Mg^{2+}.

II.B. Equilibration methods:

Before any MD simulations were carried out, the prepared proteins were minimized. During the first stage of minimization, a positional restraint (k=200 kcal/(mol·Å^2)) was applied to everything except the charge neutralizing ions and the water molecules. This stage consisted of 5,000 steepest descent minimization steps followed by 20,000 conjugate gradient steps. A second minimization stage consisted of releasing the positional restraints on the protein but keeping restraints with the same force constant on the substrates and Mg^{2+} for an additional 2000 steepest descent and 20,000 conjugate gradient minimization steps.

In equilibration scheme **1**, the system is heated to T = 300 K in the NVT ensemble while positional restraints (k=200 kcal/(mol·Å^2)) remain on everything but the neutralizing ions and water for 20 ps. Following the quick heating stage, sequential 0.1 ns NPT equilibration runs were carried out in which the positional restraints were reduced stepwise (200, 100, 50, 25, and 20 kcal/(mol·Å^2)) in each stage until all restraints were removed. Once all positional restraints were removed, a 100 ns NPT (T = 300 K, p = 1 bar) production run is carried out.

In equilibration scheme **2**, the same initial 20 ps NVT heating stage with positional restraints is carried out as in scheme **1**. Following the quick heating stage, sequential 0.1 ns NPT equilibration runs were carried out in which the positional restraints were reduced stepwise (200, 100, 50, 25, and 20 kcal/(mol·Å^2)) in each stage until the restraints were removed. At this stage,

a 20 ns equilibration run was carried out in which harmonic restraints were only applied to the Mg^{2+}-O- and Mg^{2+}-OH distances for the substrate-Mg^{2+} coordination (d_{target}=2.2 Å, k = 50 kcal/(mol·Å^2)) as well as the C-C-O-H dihedral (see Figure 1) of the substrate ($\angle_{target}$=-5.5°, k = 6.5 kcal/(mol·rad^2)). Dihedral restraints were chosen by trial and error to produce a full-width half maximum of the sampled C-C-O-H dihedral of 24° centered around -5.5°. Once the Mg^{2+}-O^- /OH distance restraints were removed, the system was equilibrated with a 100 ns NPT (T = 300 K, p = 1 bar) run with only the dihedral restraint. Finally, a 100 ns NPT production run was carried out with no restraints. Comparison of outcomes between these two equilibration schemes is provided in Sec. 3 of the main text.

Below we describe in more detail different simulation protocols that we employed. The simulation parameters (as we describe in the manuscript) are same for all the protocols.

II.B.1. Protocol 1

Step 1: Minimization 1. The system was minimized for 5,000 steps using the steepest descent method followed by 20,000 steps of the conjugate gradient method. During minimization, positional restraints were applied on the protein, SAM, inhibitor, oxygen atom of the crystallographic water, and magnesium ion with a force constant of 200 kcal mol^{-1} Å^{-2}.

Step 2: Minimization 2. The system was minimized for 2,000 steps using the steepest descent method followed by 20,000 steps of the conjugate gradient method. During minimization, positional restraints were applied on the SAM, inhibitor, oxygen atom of the crystallographic water, and magnesium ion with a force constant of 200 kcal mol^{-1} Å^{-2}.

Step 3: Heating. The system was heated to T=300 K for 20 ps using the NVT ensemble. Positional restraints were applied on the protein, SAM, inhibitor, one oxygen atom of the crystallographic water, and magnesium ion with a force constant of 200 kcal mol^{-1} $Å^{-2}$.

Step 4: Equilibration. All positional restraints were removed and the system was equilibrated for 100 ns at temperature T=300 K in the NPT ensemble.

Results: In this protocol, the hydroxyl of catecholate reorients to form an intramolecular hydrogen bond with the oxygen anion, increasing the Mg-O(H) distance nearly instantaneously.

II.B.2. Protocol 2

Step 1: Minimization 1. The system was minimized for 5,000 steps using the steepest descent method followed by 20,000 steps of the conjugate gradient method. During minimization, positional restraints were applied on the protein, SAM, inhibitor, oxygen atom of the crystallographic water, and magnesium ion with a force constant of 200 kcal mol^{-1} $Å^{-2}$.

Step 2: Minimization 2. The system was minimized for 2,000 steps using the steepest descent method followed by 20,000 steps of the conjugate gradient method. During minimization, positional restraints were applied on the SAM, inhibitor, oxygen atom of the crystallographic water, and magnesium ion with a force constant of 200 kcal mol^{-1} $Å^{-2}$.

Step 3: Heating. The system was heated to T=300 K for 20 ps in the NVT ensemble. Positional restraints were applied on the protein, SAM, inhibitor, one oxygen atom of the crystallographic water, and magnesium ion with a force constant of 200 kcal mol^{-1} $Å^{-2}$.

Step 4: Equilibration 1. The system was equilibrated at T=300 K for 20 ps in the NPT ensemble. Positional restraints were applied on the protein, SAM, inhibitor, one oxygen atom of the crystallographic water, and magnesium ion with a force constant of 100 kcal mol^{-1} $Å^{-2}$.

Step 5: Equilibration 2. The system was equilibrated at T=300 K for 20 ps in the NPT ensemble. Positional restraints were applied on the protein, SAM, inhibitor, one oxygen atom of the crystallographic water, and magnesium ion with a force constant of 50 kcal mol^{-1} $Å^{-2}$.

Step 6: Equilibration 3. The system was equilibrated at T=300 K for 20 ps in the NPT ensemble. Positional restraints were applied on the protein, SAM, inhibitor, one oxygen atom of the crystallographic water, and magnesium ion with a force constant of 25 kcal mol^{-1} $Å^{-2}$.

Step 7: Equilibration 4. The system was equilibrated at T=300 K for 20 ps in the NPT ensemble. Positional restraints were applied on the protein, SAM, inhibitor, one oxygen atom of the crystallographic water, and magnesium ion with a force constant of 20 kcal mol^{-1} $Å^{-2}$.

Step 8: Equilibration 5 or production run. All positional restraints were removed and the system was equilibrated for 100 ns at T=300 K in the NPT ensemble.

Results: Intramolecular hydrogen bond formation occurs after 0.25 ns, leading to elongation of the Mg-O(H) bond distance.

II.B.3. Protocol 3

Step 1: Minimization 1. The system was minimized for 5,000 steps using the steepest descent method followed by 20,000 steps of the conjugate gradient method. During minimization, positional restraints were applied on the protein, SAM, inhibitor, oxygen atom of the crystallographic water, and magnesium ion with a force constant of 200 kcal mol^{-1} $Å^{-2}$.

Step 2: Minimization 2. The system was minimized for 2,000 steps using the steepest descent method followed by 20,000 steps of the conjugate gradient method. During minimization,

positional restraints were applied on the SAM, inhibitor, oxygen atom of the crystallographic water, and magnesium ion with a force constant of 200 kcal mol^{-1} $Å^{-2}$.

Step 3: Heating. The system was heated to T=300 K for 20 ps in the NVT ensemble. Positional restraints were applied on the protein, SAM, inhibitor, one oxygen atom of the crystallographic water, and magnesium ion with a force constant of 200 kcal mol^{-1} $Å^{-2}$.

Step 4: Equilibration 1. The system was equilibrated at T=300 K for 20 ps in the NPT ensemble. Positional restraints were applied on the protein, SAM, inhibitor, one oxygen atom of the crystallographic water and magnesium ions with a force constant of 100 kcal mol^{-1} $Å^{-2}$.

Step 5: Equilibration 2. The system was equilibrated at T=300 K for 20 ps in the NPT ensemble. Positional restraints were applied on the protein, SAM, inhibitor, one oxygen atom of the crystallographic water, and magnesium ion with a force constant of 50 $kcalmol^{-1}$ $Å^{-2}$.

Step 6: Equilibration 3. The system was equilibrated at T=300 K for 20 ps in the NPT ensemble. Positional restraints were applied on the protein, SAM, inhibitor, one oxygen atom of the crystallographic water, and magnesium ion with a force constant of 25 kcal mol^{-1} $Å^{-2}$.

Step 7: Equilibration 4. The system was equilibrated at T=300 K for 20 ps in the NPT ensemble. Positional restraints were applied on the protein, SAM, inhibitor, one oxygen atom of the crystallographic water, and magnesium ion with a force constant of 20 $kcal.mol^{-1}$ $Å^{-2}$.

Step 8: Equilibration 5. The system was equilibrated for 50 ns at T=300 K in the NPT ensemble. NMR restraints were applied on the C-C-O-H dihedral angle (target angle= -5.5°, force constant = 6.5 $kcal.mol^{-1}.rad^{-2}$, see inset of Figure S3).

Step 9: Equilibration 6 and production run. All restraints were removed, and the system was equilibrated for 100 ns at T=300 K in the NPT ensemble.

Results: The O-H group of catecholate ion starts to flips after 2-3 ns and Mg-OH bond distance. After 76 ns the O-H groups completely flips. The H-bond distance between the H atom and the oxygen atoms of E199 are larger from the beginning.

II.B.4. Protocol 4

Step 1: Minimization 1. The system was minimized for 5,000 steps using the steepest descent method followed by 20,000 steps of the conjugate gradient method. During minimization, positional restraints were applied on the protein, SAM, inhibitor, oxygen atom of the crystallographic water, and magnesium ion with a force constant of 200 kcal mol^{-1} $Å^{-2}$.

Step 2: Minimization 2. The system was minimized for 2,000 steps using the steepest descent method followed by 20,000 steps of the conjugate gradient method. During minimization, positional restraints were applied on the SAM, inhibitor, oxygen atom of the crystallographic water, and magnesium ion with a force constant of 200 kcal mol^{-1} $Å^{-2}$.

Step 3: Heating. The system was heated to T=300 K for 20 ps in the NVT ensemble. Positional restraints were applied on the protein, SAM, inhibitor, one oxygen atom of the crystallographic water, and magnesium ion with a force constant of 200 kcal mol^{-1} $Å^{-2}$.

Step 4: Equilibration 1. The system was equilibrated at T=300 K for 20 ps in the NPT ensemble. Positional restraints were applied on the protein, SAM, inhibitor, one oxygen atom of the crystallographic water, and magnesium ion with a force constant of 100 kcal mol^{-1} $Å^{-2}$.

Step 5: Equilibration 2. The system was equilibrated at T=300 K for 20 ps in the NPT ensemble. Positional restraints were applied on the protein, SAM, inhibitor, one oxygen atom of the crystallographic water, and magnesium ion with a force constant of 50 kcal mol^{-1} $Å^{-2}$.

Step 6: Equilibration 3. The system was equilibrated at T=300 K for 20 ps in the NPT ensemble. Positional restraints were applied on the protein, SAM, inhibitor, one oxygen atom of the crystallographic water, and magnesium ion with a force constant of 25 kcal mol^{-1} $Å^{-2}$.

Step 7: Equilibration 4. The system was equilibrated at T=300 K for 20 ps in the NPT ensemble. Positional restraints were applied on the protein, SAM, inhibitor, one oxygen atom of the crystallographic water, and magnesium ion with a force constant of 20 kcal.mol^{-1} $Å^{-2}$.

Step 8: Equilibration 5. The system was equilibrated for 20 ns at T=300 K in the NPT ensemble. NMR restraints were applied on the C-C-O-H dihedral angle (target angle = -5.5°, force constant = 6.5 kcal.mol^{-1}.rad^{-2}) and on the Mg^{2+}-O^{-} and Mg^{2+}-OH bonds (target bond distance = 2.2 Å and force constant =50 kcal mol^{-1} $Å^{-2}$).

Step 9: Equilibration 6 or production run. All restraints were removed, and the system was equilibrated for 100 ns at T=300 K using the NPT ensemble. The first 30 ns of this simulation was discarded for production analysis.

Results: The bidentate coordination of Mg^{2+} is preserved for the entire 70 ns simulation (step 9). However, the active site is somewhat disrupted: we observe long distances between E199 and the hydrogen of catecholate. We also see unusually long C-O distances between the methyl group of SAM and the anionic oxygen of catecholate.

II.B.5. Protocol 5

Step 1: Minimization 1. The system was minimized for 5,000 steps using the steepest descent method followed by 20,000 steps of the conjugate gradient method. During minimization, positional restraints were applied on the protein, SAM, inhibitor, oxygen atom of the crystallographic water, and magnesium ion with a force constant of 200 kcal mol^{-1} $Å^{-2}$.

Step 2: Minimization 2. The system was minimized for 2,000 steps using the steepest descent method followed by 20,000 steps of the conjugate gradient method. During minimization, positional restrained were applied on the SAM, inhibitor, oxygen atom of the crystallographic water, and magnesium ion with a force constant of 200 kcal mol^{-1} $Å^{-2}$.

Step 3: Heating. The system was heated to T=300 K for 20 ps in the NVT ensemble. Positional restraints were applied on the protein, SAM, inhibitor, one oxygen atom of the crystallographic water, and magnesium ion with a force constant of 200 kcal mol^{-1} $Å^{-2}$.

Step 4: Equilibration 1. The system was equilibrated at T=300 K for 20 ps in the NPT ensemble. Positional restraints were applied on the protein, SAM, inhibitor, one oxygen atom of the crystallographic water, and magnesium ion with a force constant of 100 kcal mol^{-1} $Å^{-2}$.

Step 5: Equilibration 2. The system was equilibrated at T=300 K for 20 ps in the NPT ensemble. Positional restraints were applied on the protein, SAM, inhibitor, one oxygen atom of the crystallographic water, and magnesium ion with a force constant of 50 $kcalmol^{-1}$ $Å^{-2}$.

Step 6: Equilibration 3. The system was equilibrated at T=300 K for 20 ps in the NPT ensemble. Positional restrained were applied on the protein, SAM, inhibitor, one oxygen atom of the crystallographic water, and magnesium ion with a force constant of 25 kcal mol^{-1} $Å^{-2}$.

Step 7: Equilibration 4. The system was equilibrated at T=300 K for 20 ps in the NPT ensemble. Positional restraints were applied on the protein, SAM, inhibitor, one oxygen atom of the crystallographic water, and magnesium ion with a force constant of 20 kcal.mol^{-1} Å^{-2}.

Step 8: Equilibration 5. The system was equilibrated for 20 ns at T=300 K in the NPT ensemble. NMR restraints were applied on the C-C-O-H dihedral angle (target angle= -5.5°, force constant = 6.5 kcal.mol^{-1}.rad^{-2}) and on the Mg^{+2}-O^{-} and Mg^{+2}-OH bonds (target bond distance =2.2 Å and force constant =50 kcal mol^{-1} Å^{-2}).

Step 9: Equilibration 6. The system was equilibrated for 100 ns at T=300 K in the NPT ensemble. NMR restraints were applied only on the C-C-O-H dihedral (target angle= -5.5°, force constant = 6.5 kcal.mol^{-1}.rad^{-2}).

Step 10: Equilibration 7 or production run. All restraints were removed and the system was equilibrated for 100 ns at T=300 K in the NPT ensemble.

Results: The bidentate coordination of Mg^{2+} is preserved for the entire 100 ns simulation.

II.C. Determining the force constant for the dihedral angle NMR restraint

We carried out several simulations to identify the appropriate minimum force constant for the C-C-O-H dihedral angle restraint. This procedure started with minimization, NVT heating with positional restraints, and NPT equilibration with gradual removal of positional restraints. We collected a target C-C-O-H dihedral distribution based on a case where a bidentate configuration between the substrate and Mg^{2+} were always preserved (see inset of the Figure S3 for the C-C-O-H dihedral angle). The full width half maximum (FWHM) of the sampled dihedral distribution is 23°.

Next, we simulated the system with the C-C-O-H angle restraint during the production run in order to confirm that the substrate remained in a bidentate configuration with the Mg^{2+} ion. In order to reproduce the dihedral angle distribution where no restraints were used, we ran several simulations with different force constants with the C-C-O-H target angle=-5.5°. As we can see in Table S2, the FWHM is 24° for both the systems with the force constant 5 and 6.5 $kcal \cdot mol^{-1} \cdot rad^{-2}$. We also considered representative bond distances in the active site. Short non-bonded C-O distances are best preserved in the simulation with a 6.5 $kcal \cdot mol^{-1} \cdot rad^{-2}$ force constant.

II.D. Umbrella sampling restraint details

The Mg^{2+}-OH distance was sampled from 2.0 to 4.5 A in 0.1 Å increments, and the C-C-O-H dihedral (see Figure S3 inset) was sampled from 0° to 180° in 5° increments. Initial configurations were generated for each window from a multistep process. First, an initial configuration was selected from the long direct MD production run. Next, a series of seven sequential restrained minimizations and short dynamics runs were carried out. The snapshot from the production run MD was used as a starting point to generate the first configuration ($\angle_{CCOH}$=0°, $d_{Mg\text{-}OH}$=2.2 Å) through a multi-step process. In this equilibration, a 2000 step steepest descent minimization with positional restraints on the protein and substrates (k=200 kcal/(mol·Å^2)) was carried out followed by NPT (T = 300 K, p = 1 bar) equilibration for 300 ps using a 200 kcal/(mol·rad^2) force constant on the dihedral and 500 kcal/(mol·Å^2) on the bond distance. The resulting structure was then visually inspected to verify that the target distance and dihedral were satisfied without distorting the surrounding protein and substrate environment. The outcome of this simulation was also used as the starting point for the next coarse minimization and equilibration window ($\angle_{CCOH}$=30°, $d_{Mg\text{-}OH}$=2.2 Å). The minimization, equilibration, and

inspection procedure was repeated sequentially for the other five windows ($\angle_{CCOH}$=60°, $d_{Mg\text{-}OH}$=2.2 Å; $\angle_{CCOH}$=90°, 120°, 150°, 170° at $d_{Mg\text{-}OH}$=3.5 Å).

The resulting seven structures from the coarse windows were used as starting points for the 37x26 windows on the two-dimensional free energy surface ($\angle_{CCOH}$=0° used for 0-20°; $\angle_{CCOH}$=0° for 25-45°; $\angle_{CCOH}$=60° for 50-75°; $\angle_{CCOH}$=90° for 80-105°; $\angle_{CCOH}$=120° for 110-135° , $\angle_{CCOH}$=150° for 140-160°, and $\angle_{CCOH}$=170° for 165-180°). For each window, a 5000 step steepest descent minimization with positional restraints on the protein and substrates (k=200 kcal/(mol·Å^2)) was carried out followed by NPT (T = 300 K, p = 1 bar) equilibration for 100 ps using a 200 kcal/(mol·rad^2) force constant on the dihedral and 500 kcal/(mol·Å^2)) on the bond distance and bond and dihedral distributions were collected during a 200 ps production run in the NPT ensemble (T = 300 K, p = 1 bar) for a total of 288 ns simulation for each substrate. Unbiased free energies were obtained from the sampled distributions using the weighted histogram analysis method (WHAM)[3-4] using the Grossfield lab WHAM software package[5]. The Amber code does not multiply force constants for distances by ½ while the WHAM package does, so force constants were rescaled accordingly for analysis. Distributions were visually inspected to confirm that distributions were overlapping between windows, and the force constants for dihedrals and distances were obtained by tuning the balance between overlapping distributions and sufficiently sampling the target distance and dihedral.

References

1. Allnér, O.; Nilsson, L.; Villa, A., Magnesium Ion–Water Coordination and Exchange in Biomolecular Simulations. *J. Chem. Theory Comput.* **2012,** *8*, 1493-1502.
2. Li, P.; Song, L. F.; Merz Jr, K. M., Parameterization of Highly Charged Metal Ions Using the 12-6-4 Lj-Type Nonbonded Model in Explicit Water. *J. Phys. Chem. B* **2014,** *119*, 883-895.

3. Souaille, M.; Roux, B. t., Extension to the Weighted Histogram Analysis Method: Combining Umbrella Sampling with Free Energy Calculations. *Comput. Phys. Commun.* **2001,** *135*, 40-57.
4. Kumar, S.; Rosenberg, J. M.; Bouzida, D.; Swendsen, R. H.; Kollman, P. A., The Weighted Histogram Analysis Method for Free-Energy Calculations on Biomolecules. I. The Method. *J. Comput. Chem.* **1992,** *13*, 1011-1021.
5. Grossfield, A. "Wham: The Weighted Histogram Analysis Method", Version 2.0.9 http://membrane.urmc.rochester.edu/content/wham (accessed 06/15/2015).
