# Supplementary material for: Computational Investigation of the Interplay of Substrate Positioning and Reactivity in Catechol O-Methyltransferase
Source: PLoS One. 2016 Aug 26;11(8):e0161868. doi: 10.1371/journal.pone.0161868 (PMC5001633; doi:10.1371/journal.pone.0161868)
Supplement: S2 Text — (PDF) [file pone.0161868.s023.pdf]

II. Free energy differences between PM6 and AM1/d

II.A. Estimating enthalpic differences between PM6 and B3LYP/6-311++G*

Here, we carried out calculations to identify how energetic profiles might differ between QM (DFT) and SQM approaches in the gas phase as well as in implicit solvent environments meant to approximately model a protein (ε=10) and aqueous solution (ε=78.4). We first considered the reaction energies for both trimethylsulfonium (TMS) cation, a common model for SAM employed in the literature[1-3], and the full SAM substrate with catecholate anion in the absence of Mg^{2+}. Generally, B3LYP reaction energies for either TMS or SAM are consistently more exothermic than PM6 energetics by around 20 kcal/mol (Supporting Information Table S7). We also calculated the unconstrained methyl transfer in a protein-like dielectric at the B3LYP and PM6 levels of theory using nudged elastic band within the B3LYP calculations and repeating single point energies with PM6. We found the PM6 barrier to be enhanced by around 10 kcal/mol with respect to B3LYP (Supporting Information Figure S12).

Finally, we considered a more realistic comparison directly based on the free energy surfaces computed from PM6/MM. Here, we extracted representative snapshots along the methyl transfer coordinate of all residues in the SMgL SQM region complete with hydrogen capping and carried out constrained optimizations in a protein-like dielectric (see Computational Details and Supporting Information). The resulting enthalpic barriers were comparable for both PM6 and B3LYP although B3LYP reaction energetics remained a few kcal/mol slightly more favorable for the SMgL model system (Supporting Information Figure S13). Overall, these results support the interpretation and conclusions of the PM6/MM simulations, although further comparisons to free energies obtained directly from DFT QM/MM are ongoing in our lab.

Comparison of SQM and QM methods were carried out. These calculations included the extraction of models of the SQM region from SQM/MM dynamics. In these cases, we used constrained optimizations with S-C and C-O distances as well as Ca carbon atom positions of protein residues held fixed to their values from SQM/MM dynamics in order to obtain an approximate minimum energy path for comparison of DFT and SQM methods. DFT calculations employed the 6-311++G* localized basis set with the TeraChem[4-5] code. The MOPAC simulation package[6-7] was used for PM6 geometry optimizations. Benchmarks on methyl transfer model reactions were used to identify systematic deviations of SQM from QM results (see Supporting Information). These comparisons were made in the gas phase or with an implicit solvent model with a dielectric intended to very approximately represent a protein environment (ε=10) or liquid water (ε=78.4). True proteins have variable dielectric constants that may be lower, but these calculations were intended solely to approximate a point intermediate between gas phase and aqueous solvent. TeraChem implicit solvent calculations employed the conductor-like screening model (COSMO)[8-9], as did the MOPAC PM6 calculations. Good agreement of B3LYP and PM6 for the largest Mg^{2+}-containing SQM regions was observed, which is perhaps not surprising given that PM3 has been reported to give a good estimates of the hydration free energy of magnesium cation[10].

Semi-empirical methods may fail to correlate well to density functional theory results, particularly for the estimation of bond breaking events and calculation of transition states. We computed several reaction energies and transition states to compare PM6 and B3LYP/6-311++G*. The B3LYP/6-311++G* results were obtained with TeraChem, while the PM6 calculations were carried out with MOPAC. Our sample calculations were run both in the gas

phase and in a COSMO polarizable continuum model with dielectric modeling water (ε=78.4) and one approximating a protein environment (ε=10.0).

Model methyl donors were S-adenosyl methionine (SAM) or trimethylsulfonium (TMS). The methyl acceptor was catecholate. End points of the reactions were optimized in the respective programs. The reaction energetics are consistently more favorable at the B3LYP level than they are at the PM6 level (Table S5).

We also computed the reaction path for methyl transfer from SAM to catecholate with a dielectric value of 10. We obtained the converged path with nudged elastic band in TeraChem at the B3LYP/6-311++G* level of theory and then computed single points at the PM6 level of theory. Here, the reaction energetics again are underestimated by PM6 with respect to B3LYP. PM6 also overestimates the B3LYP-computed barrier by several kcal/mol (Figure S13).

Representative snapshots were extracted at target values of the reaction coordinate (d(S-C)-d(C-O)) from the umbrella sampling runs with all SMgL residues included in the model (D141, K144, D169, N170, E199, water, Mg^{2+}, SAM, catecholate). All dangling bonds were passivated with hydrogen using pymol. Constrained optimizations were carried out in which S, C, O positions of the transferring atoms were held fixed as were the Cα carbons atoms of any protein residues in the SMgL model (D141, K144, D169, N170, E199). These optimizations were carried out in MOPAC for the PM6 level of theory and in TeraChem for B3LYP/6-311++G*. Overall barrier heights are comparable for the two methods at around 22-23 kcal/mol, while B3LYP still demonstrates more favorable reaction energetics than PM6 by about 7 kcal/mol.

References

1. Zheng, Y. J.; Bruice, T. C., A Theoretical Examination of the Factors Controlling the Catalytic Efficiency of a Transmethylation Enzyme: Catechol O-Methyltransferase. *J. Am. Chem. Soc.* **1997**, *119*, 8137-8145.
2. Lameira, J.; Bora, R. P.; Chu, Z. T.; Warshel, A., Methyltransferases Do Not Work by Compression, Cratic, or Desolvation Effects, but by Electrostatic Preorganization. *Proteins: Struct., Funct., Bioinf.* **2015**, *83*, 318-330.
3. Rod, T. H.; Ryde, U., Quantum Mechanical Free Energy Barrier for an Enzymatic Reaction. *Phys. Rev. Lett.* **2005**, *94*, 138302.
4. Petachem. http://www.petachem.com (accessed Jul. 27, 2015).
5. Ufimtsev, I. S.; Martinez, T. J., Quantum Chemistry on Graphical Processing Units. 3. Analytical Energy Gradients, Geometry Optimization, and First Principles Molecular Dynamics. *J. Chem. Theory Comput.* **2009**, *5*, 2619-2628.
6. Maia, J. D. C.; Urquiza Carvalho, G. A.; Mangueira, C. P.; Santana, S. R.; Cabral, L. A. F.; Rocha, G. B., Gpu Linear Algebra Libraries and Gpgpu Programming for Accelerating Mopac Semiempirical Quantum Chemistry Calculations. *J. Chem. Theory Comput.* **2012**, *8*, 3072-3081.
7. Stewart, J. J. P., Mopac2012, Stewart Computational Chemistry, Version 15.156l.
8. Klamt, A., Conductor-Like Screening Model for Real Solvents: A New Approach to the Quantitative Calculation of Solvation Phenomena. *J. Phys. Chem.* **1995**, *99*, 2224-2235.
9. Liu, F.; Luehr, N.; Kulik, H. J.; Martínez, T. J., Quantum Chemistry for Solvated Molecules on Graphical Processing Units Using Polarizable Continuum Models. *J. Chem. Theory Comput.* **2015**, *11*, 3131-3144.
10. Furuki, T.; Sakurai, M.; Inoue, Y., An Application of the Reaction Field Theory to Hydrated Metal Cations in the Framework of the Mndo, Am1, and Pm3 Methods. *J. Comput. Chem.* **1995**, *16*, 378-384.
