# Supplementary material for: Computational Investigation of the Interplay of Substrate Positioning and Reactivity in Catechol O-Methyltransferase
Source: PLoS One. 2016 Aug 26;11(8):e0161868. doi: 10.1371/journal.pone.0161868 (PMC5001633; doi:10.1371/journal.pone.0161868)
Supplement: S3 Text — (PDF) [file pone.0161868.s024.pdf]

III. Description of MMPBSA Theory

In this method, the binding free energy is decomposed into the relative free energy of the solvated receptor(protein)-ligand complex and the separated, solvated ligand and receptor:

$$\Delta G_{\text{binding,solvated}} = \Delta G_{\text{complex,solvated}} - \Delta G_{\text{receptor,solvated}} - \Delta G_{\text{ligand,solvated}} \quad . \tag{1}$$

Each free energy change in eq. 1 is comprised of the sum of several terms:

$$\Delta G_{\text{solvated}} = \Delta E_{\text{MM}} + \Delta G_{\text{pol}} + \Delta G_{\text{nonpol}} + \Delta G_{\text{dispersion}} - T\Delta S \,, \tag{2}$$

which are i) the molecular mechanics energy, ii) the polar solvation free energy estimated from the Poisson-Boltzmann(PB)[1] or Generalized Born (GB)[2] models, iii) the nonpolar solvation energy obtained as a function of the solvent accessible surface area, iv) a cavity dispersion term, and v) an estimate of the solute entropy. Typically[3], the solute entropy term is neglected, as its inclusion is more sensitive to incomplete sampling errors than other terms. The molecular mechanics energy (ΔE_{MM}) is further decomposed into an electrostatic (elst) component and a van der Waals (vdW) contribution. Within MMPBSA, rigid binding is assumed and configurational effects are averaged over several hundred snapshots (see Computational Details in main text) and therefore any differences in internal, bonded terms between the complex, receptor, and ligand are zero. The total binding free energy may be grouped into a solvent contribution:

$$\Delta G_{\text{solv}} = \Delta G_{\text{disper}} + \Delta G_{\text{pol}} + \Delta G_{\text{nonpol}} \quad , \tag{3}$$

and a gas phase contribution:

$$\Delta G_{\text{gas}} = \Delta G_{\text{elst}} + \Delta G_{\text{vdW}} \quad . \tag{4}$$

Gas phase binding energies are likely to be strongly favorable, while the solvation contribution is likely to be repulsive, as it includes the energetic cost of desolvating the ligand.

References

1. Sharp, K. A.; Honig, B., Calculating Total Electrostatic Energies with the Nonlinear Poisson-Boltzmann Equation. *J. Phys. Chem.* **1990**, *94*, 7684-7692.
2. Tsui, V.; Case, D. A., Theory and Applications of the Generalized Born Solvation Model in Macromolecular Simulations. *Biopolymers* **2000**, *56*, 275-291.
3. Hou, T.; Wang, J.; Li, Y.; Wang, W., Assessing the Performance of the Mm/Pbsa and Mm/Gbsa Methods. 1. The Accuracy of Binding Free Energy Calculations Based on Molecular Dynamics Simulations. *J. Chem. Inf. Model.* **2011**, *51*, 69-82.
